# The Role of Striatum in Controlling Waiting during Reactive and Self-Timed Behaviors

**DOI:** 10.1523/JNEUROSCI.1820-24.2025

**Published:** 2025-02-14

**Authors:** Qiang Zheng, Yujing Liu, Yue Huang, Jiaming Cao, Xuanning Wang, Jianing Yu

**Affiliations:** ^1^State Key Laboratory of Membrane Biology, School of Life Sciences, Peking University, Beijing 100871, China; ^2^School of Life Sciences, Peking University, Beijing 100871, China; ^3^Peking-Tsinghua Center for Life Sciences, Academy for Advanced Interdisciplinary Studies, Peking University, Beijing 100871, China; ^4^IDG/McGovern Institute for Brain Research, Peking University, Beijing 100871, China; ^5^Chinese Institute for Brain Research, Beijing 102206, China

**Keywords:** anticipation, basal ganglia, motor cortex, reaction time, vigor

## Abstract

The ability to wait before responding is crucial for many cognitive functions, including reaction time (RT) tasks, where one must resist premature actions before the stimulus and respond quickly once it is presented. However, the brain regions governing waiting remain unclear. Using localized excitotoxic lesions, we investigated the roles of the motor cortex (MO) and sensorimotor dorsolateral striatum (DLS) in male rats performing a conditioned lever-release task with variable delays. Neural activity in both MO and DLS showed similar firing patterns during waiting and responding periods. However, only bilateral DLS lesions caused a sustained increase in premature (anticipatory) responses, whereas bilateral MO lesions primarily prolonged RTs. In a self-timing version of the task, where rats held a lever for a fixed delay before releasing it, DLS lesions caused a leftward shift in response timing, leading to persistently greater premature responses. These waiting deficits were accompanied by reduced motor vigor, such as slower reward-orienting locomotion. Our findings underscore the critical role of the sensorimotor striatum in regulating waiting behavior in timing-related tasks.

## Significance Statement

Waiting is essential for the temporal control of actions, as many cognitive behaviors—whether stimulus-driven or internally planned—require withholding a response until the appropriate time. However, the neural substrates of waiting remain less understood. Using targeted lesions, we identified the dorsolateral striatum as a crucial region for waiting in both reaction time (RT) and self-timing tasks. Lesions in this area caused a persistent increase in premature responses across tasks. In contrast, motor cortex lesions, despite its neurons showing similar activity patterns to the striatum during waiting, did not result in a lasting increase in premature responses; instead, they led to a long-term increase in RT.

## Introduction

Waiting before responding is a fundamental executive function of the brain and is crucial for adaptive behavior. In simple reaction time (SRT) behavior, subjects must hold off on a planned action until the stimulus signal prompts them to respond. For instance, a sprinter initiates a run when the starter's gun is fired or a subject releases a key upon perceiving a visual signal. The time taken from the onset of the stimulus to the initiation of the motor response, known as reaction time (RT), typically spans several hundred milliseconds (Evarts, 1966; Ratcliff and Murdock, 1976; Brown and Robbins, 1991). RT encapsulates the delays accumulated across multiple stages of neural processing (Donders, 1969) and is a valuable measure of the subject's physical and cognitive abilities (Blokland, 1998; Laubach et al., 2015).

Cognitive control during SRT tasks is best demonstrated during the foreperiod (FP)—the waiting or delay phase. During the FP, a subject must inhibit the urge to respond, which is regarded as a type of inhibitory control. Failing to do so leads to premature responses. Subjects may anticipate the stimulus timing, preparing to act, or even acting before the signal appears (Ollman and Billington, 1972; Kornblum, 1973; Niemi and Näätänen, 1981). Once the stimulus signal is delivered, the motor system must rapidly engage to generate a fast reaction. These two processes—waiting and reacting—represent competing processes that the brain must balance to achieve optimal performance.

The urge to respond quickly is often accompanied by an increase in premature responses, suggesting the existence of a speed-accuracy trade–off (Luce, 1991). Several theoretical frameworks have been proposed to explain SRT behavior. The “deadline” model posits that responses are triggered by stimulus only if the sum of the FP and the response latency is shorter than a random deadline variable; otherwise, when the deadline is reached, the subject responds regardless of the stimulus (Ollman and Billington, 1972). Another theory views RT behavior as a competition between an internal clock and a signal detector, with the first to reach the response generator dictating the response (Kornblum, 1973). These frameworks suggest that waiting is an active process governed by an internal response model instructing subjects when to respond, regardless of the signal. Thus, premature responses are also referred to as “anticipatory” responses. However, the precise neural basis of this internal model and its relationship to the response generator remain unclear. This study seeks to identify the neural substrates involved in these processes, focusing on the corticostriatal system, given its direct connection to motor output.

In rodents, a significant portion of the frontal cortex is dedicated to representing body movement, forming the motor cortex (MO), where low-amplitude current injection induces movements in various body parts (Donoghue and Wise, 1982; Neafsey et al., 1986; Wise and Donoghue, 1986; Brown and Teskey, 2014). MO lesions can result in deficits in skillful movement and changes in handedness, though recovery has often been observed over time (Castro, 1972; Whishaw et al., 1986; Lemke et al., 2019; Nicholas and Yttri, 2024). The MO contains neurons active during or before conditioned responses (Evarts, 1966; Narayanan and Laubach, 2009). Some studies have reported no significant effect of MO inactivation on RT (Martin et al., 1993), whereas others have shown a temporary or lasting increase (Brown et al., 1991; Matsumura et al., 1991; Baunez et al., 1998; Smith et al., 2010; Nicholas and Yttri, 2024).

The primary MO sends dense axonal fibers to the dorsolateral portion of the striatum (Ebrahimi et al., 1992; Rouiller et al., 1993), also known as the dorsolateral striatum (DLS), a key input region of the basal ganglia (McGeorge and Faull, 1989; Wu et al., 2000; Hunnicutt et al., 2016). Many studies have examined the role of DLS in RT behaviors, focusing on manipulating the neurotransmitter, particularly dopamine. Depleting dopamine in the dorsal striatum has been shown to produce complex behavioral impairments, including slowing down the RT in some animals and increasing premature responses in most animals (Amalric and Koob, 1987; Amalric et al., 1995). A handful of studies have performed lesions in DLS in various RT paradigms and reported no significant change in RT (Hauber and Schmidt, 1994; Brasted et al., 1998). One study mentioned that DLS lesions introduced more premature errors in a RT task involving a sustained nose poke during the delay (Brasted et al., 1998).

Previous studies have yet to examine MO and DLS using comparable perturbations within the same RT task, making it difficult to integrate these observations into a cohesive mechanism. In this study, we combined electrophysiological recordings and lesions to contrast the roles of the MO and DLS during an SRT task in which rats were trained to hold a lever for variable delays before responding to a stimulus signal (Amalric and Koob, 1987). Behavioral performance was assessed following unilateral or bilateral excitotoxic lesions in either the MO or DLS, addressing the long-term necessity of these regions (Vaidya et al., 2019) and examining their dissociable effects. We found that the DLS, but not MO, plays a key role in suppressing premature responses during the SRT task. Moreover, the DLS is also critical for prolonging waiting time in a self-timing task.

## Materials and Methods

### Subjects

All animal procedures complied with the animal care standards set forth by the US National Institutes of Health and were approved by the Institutional Animal Care and Use Committee of Peking University. Male adult Brown Norway or Long–Evans rats, aged at least 3 months and weighing 250–400 g prior to water restriction and behavioral training, were obtained from Vita River. The rats were housed individually or in pairs/groups of 2–3 on a 12 h reverse light/dark cycle, with postsurgery housing restricted to individual cages. During water restriction, water was earned through behavioral sessions, with supplemental water provided at least 3 h after a session if intake was <12 ml. Rats' body weights were maintained at ∼80% of their *ad libitum* feeding weight. Training was conducted 6 d per week, with unrestricted water access on the seventh day.

### Apparatus

Behavioral sessions were conducted in five operant conditioning chambers (Med Associates) integrated with Bpod State Machines (Sanworks). Each chamber was equipped with a retractable lever positioned 3.8–4.3 cm above the floor, along with a house light mounted near the ceiling and a lever light located either directly above or adjacent to the lever. A reward port, situated 26 or 32 cm opposite the lever, was controlled by the Bpod State Machine. Upon a correct response, the reward port illuminated, signaling a state transition in the Bpod system, and delivered a liquid reward (60 µl of 10% sucrose solution) following a nose poke at the port. Auditory stimuli were presented via a speaker mounted above the lever, delivering a 250 ms, 70 dB, 2,900 Hz pure tone. Additionally, a blue LED was positioned above the lever. For behavioral experiments, the conditioned stimulus signaling lever release consisted of a combination of the tone and LED flash, both lasting 250 ms. For electrophysiological recordings, only the tone served as the conditioned stimulus. A camera (Hikvision or Daheng Imaging) was positioned outside each chamber to capture a side view of the lever-pressing response at 50 Hz. In one chamber, an additional camera was positioned to record a bird's-eye view of locomotor behavior.

### Behavioral training

Behavioral training for the SRT and self-timing tasks followed a multistep protocol based on a program provided by Drs. Marcelo Caetano and Mark Laubach. Each session lasted up to 90 min, and throughout all lever-related steps (2–7), the lever remained extended until the session's end, ensuring self-initiated lever presses as follows:

1. Conditioning to Tone: Rats were trained to respond to a 250 ms pure tone delivered after a random delay (mean, 30 s). The reward port illuminated with tone delivery, and poking the port after the stimulus triggered a reward. Early port entries reset the delay.
2. Lever Press Training: Rats learned to press the lever, which triggered the tone and illuminated the reward port. A subsequent port poke then delivered the reward. Initially random, lever presses became associated with reward delivery to the rats.
3. Lever Release Training: In this phase, rats were trained to release the lever after pressing it. The release triggered the tone and port light, followed by a reward upon port entry.
4. Hold and Release Training: Rats were trained to hold the lever for progressively longer FPs, starting at 0.25 s and increasing by 0.1 s after three consecutive correct responses, up to 1.5 s. Releasing the lever after the FP, signaled by a 250 ms tone, was rewarded, while premature or late releases (after a 2 s window) resulted in a 3–5 s timeout. Timeout was reset if the lever was pressed again during the timeout.
5. Rapid Response Training: The FP increased progressively, following the same pattern as in “Hold and Release Training” but the response window was progressively reduced to 0.6 s after consecutive correct responses.
6. SRT Task: Once rats achieved >70% accuracy in the last step, they were tested with randomly presented FPs of 0.5, 1.0, and 1.5 s, with a response window of 0.6 s. In recent experiments, a subset of trials (10%) was designated as probe trials, during which no stimulus was presented, and lever release produced no outcome.
7. Self-Timing Task: Rats transitioned to a 1.0 s fixed FP protocol with cued and uncued trials. In cued trials, a tone followed the FP, as in the SRT task. In uncued trials, in the absence of a trigger signal, lever release within 1.0 s after the FP was rewarded. A tone followed each correct release. The proportion of uncued trials increased to 90%, encouraging the use of time estimation strategies.

### Surgery

Rats were anesthetized with 4% isoflurane for induction and maintained at 1.5–2% throughout the procedure. Quinolinic acid (0.1 M, P63204, Sigma-Aldrich) was freshly prepared in phosphate-buffered saline (PBS), pH 7.4, immediately before injection. Excitotoxic lesions were made by infusing the toxin (50–100 nl/min) through a pulled glass pipette into the brain at the coordinates detailed in Table 1. For unilateral lesions, injections were made contralateral to the preferred paw. Rats received lesions in the unilateral MO (contralateral to the preferred paw), bilateral MO (bMO), unilateral DLS (contralateral to the preferred paw), and bilateral DLS (Extended Data Table 4-1) or underwent sham surgery. Sham surgeries included all procedural steps (skin incision, skull cleaning, craniotomy) with either PBS injection (*N* = 9) or partial craniotomy (*N* = 2).

**Table 1.** Coordinates of toxin injections

Region	AP (mm)	ML (mm)	DV (mm)	Dose (nL)
MO	0	2	1.6	400
	0.2	3.5	1.6	400
	1.2	3	1.6	400
	2.5	2.5	1.6	400
	3	3	1.6	400
	3.5	2	1.6	400
DLS	1.5	3.8	4	100
	1.5	3.8	4.4	150
	1.5	3.8	4.8	200
	0.5	4	4	100
	0.5	4	4.4	150
	0.5	4	4.8	200

To assess the extent of MO lesions, 10 rats with bMO lesions received additional spinal injections before perfusion. Under deep isoflurane anesthesia, rats were placed in a stereotaxic frame, and a midline incision was made to expose the C4–T2 vertebrae. The spinal column was stabilized with a rat spinal adaptor (World Precision Instruments), and the meninges at C4–C6 were gently resected. Red retrograde beads (Lumafluor) were injected into the spinal cord using a pulled pipette. A total of 400 nl of beads was infused into each of the C4, C5, and C6 spinal segments at ±1 mm lateral to the midline and 1.6 mm ventral to the cord surface. Brain sections (80 µm thick) were scanned using a microscope slide scanner (OLYMPUS VS120) with a 10× objective, and bead location was manually traced using ImageJ (Fig. 4).

### Analysis of behavioral data

Press, release, and tone times were recorded using MED and analyzed with custom MATLAB routines (MathWorks). RT was defined as the latency between the tone and lever release, excluding latencies shorter than 0.1 s (too brief for reactive responses) or longer than 2 s. Outliers were removed if they exceeded 10 times the median absolute deviation (MAD) from the median, using MATLAB's rmoutliers function. RT distributions were computed by pooling both correct and late responses, with the median representing the session or condition (e.g., prelesion). The probability density function of hold duration was estimated using kernel density estimation (ksdensity in MATLAB) with a bandwidth of 0.075 and a bin width of 0.05 s. Event timing was recorded with a temporal resolution of 10 ms.

For postlesion analyses, sessions with fewer than 50 responses were combined with subsequent sessions to ensure sufficient sample size for statistical analysis (e.g., Figs. 5*A*,*B*, 6*A*–*D*, 7, 8*B*–*E*, 9*B*–*F*). To compare response distributions before a lesion, immediately after a lesion, and after extensive postlesion training (e.g., Figs. 5*C*,*D*, 6*E*, 8*D*,*F*,*G*), the final prelesion sessions for each rat were combined until the trial count for each FP exceeded 500 (Pre). Responses from full sessions were included (i.e., responses from sessions were combined until the response number exceeds 500). Early postlesion sessions were combined until the trial count for each FP exceeded 200 (Post_Early_), and final postlesion sessions were combined similarly (Post_Late_).

An anticipation function *G*(*t*) was computed based on the deadline model (Ollman and Billington, 1972) in 10 ms intervals from 0 to 1.5 s. For each time interval, the denominator of *G*(*t*) represents the number of trials with FP equal to or longer than the given time interval, while the numerator represents the cumulative count of responses up to that time interval. By definition, these are the premature responses. The function *G*(*t*) was defined as follows for each time interval:

$$\begin{array}{rl} G(t)= & \frac{N_{1}\cdot F_{1}(t)+N_{2}\cdot F_{2}(t)+N_{3}\cdot F_{3}(t)}{N_{1}+N_{2}+N_{3}},\;\mathrm{for}\;t\;<\;0.5\;\\ G(t)= & \frac{N_{2}\cdot F_{2}(t)+N_{3}\cdot F_{3}(t)}{N_{2}+N_{3}},\;\mathrm{for}\;0.5\;\leq \;t\;<\;1\;\\ G(t)= & \frac{N_{3}\cdot F_{3}(t)}{N_{3}},\;\mathrm{for}\;1\;\leq \;t\;<1.5\;, \end{array}$$

where $N_{i}(i=1,\;2,\;3)$ is the number of trials with FPs of 0.5, 1, 1.5 s. $F_{i}(t)(i=1,\;2,\;3)$ denotes the fraction of premature responses that occurred at or before time t for each trial type.

### Locomotor and paw movement analysis

The trigger stimulus activated an LED, which served as a reference signal to synchronize video frames with behavioral events. Using LED onsets recorded by the video computer and trigger signals from the behavioral computer, video timestamps were aligned to the behavioral time domain. Based on this alignment, raw videos were segmented into short clips that spanned 2 s before the lever press and 2 s after the lever release. From these clips, frames that captured key aspects of paw movement, such as paw lifting and lever pressing, were extracted. A deep neural network model, trained using the DeepLabCut package (Mathis et al., 2018), was used to accurately classify and track the paws and digits in these frames. Specifically, we focused on tracking Digit 4 or 5 of the rat's paw, constructing a trajectory that was manually refined using custom MATLAB applications. Paw trajectory tracking began when the paw lifted off the floor and continued for at least 1 s after the lever press. The speed of each reaching movement was calculated by integrating the total length of the trajectory from lift-off to the highest point of the paw's path and dividing this length by the time it took to reach that point. To track locomotor activity from the top-view videos, the right ear of the animal was tracked using the same method.

To account for drift in the camera’s field of view across sessions, a cross-correlation method was employed to correct for these changes. The lever-reaching trajectories were then shifted by aligning endpoints to prevent differences in paw landing positions on the lever from artificially reducing trajectory correlations (this refinement was not applied to locomotor movements from top-view videos). Each trajectory was mapped onto a grid and converted into a binary matrix for analysis, which was then smoothed using a 2D Gaussian filter (width = 25 pixels). Trajectory correlation was determined by calculating the dot product of their respective matrices. To measure trajectory similarity, we computed the median correlation from all pairwise comparisons within the same condition (e.g., Pre~Pre) or between different conditions (e.g., Pre~Post_Late_).

### Reward retrieval duration

The reward retrieval duration was defined as the time it took for the rat to move from a correct lever release to a nose poke at the reward port, which was located 26 or 32 cm away on the opposite side of the behavioral box. Outliers were removed if they exceeded 10 times the MAD from the median, using MATLAB's rmoutliers function. To evaluate how reward retrieval duration changed across sessions due to lesions and postlesion training, any postlesion session with fewer than 30 responses was combined with subsequent sessions until at least 30 responses were collected for a session.

### Electrophysiological recordings

Chronic electrophysiological recordings were conducted on trained rats in an operant box that was slightly larger but otherwise identical to the training setup. For primary MO recordings, electrodes were positioned 1–2 mm anterior and 2.3–3 mm lateral to the bregma. Recordings were taken from eight rats using either 16- or 32-channel fixed nichrome microwire arrays (Bio-Signal; *N* = 3), custom-made movable 32-channel nitinol tetrodes (Kedou Brain Computer Technology; *N* = 4), or 32-channel silicon probes mounted on a movable drive (Diagnostic Biochips; *N* = 1; Vöröslakos et al., 2021). For fixed arrays, electrodes were initially inserted 1.8 mm below the brain surface. For movable electrodes, the initial insertion depth was 1.2 mm, and electrodes were advanced by 25–50 µm after each recording session until the electrode tips reached the bottom of Layer 6, where neural activity became sparse. For striatum (DLS) recordings, electrodes were centered at 0.5 mm anterior and 4.0 mm lateral to the bregma. Recordings were made in nine rats using either 16- or 32-channel fixed microwire arrays (*N* = 7) or 32-channel silicon probes mounted on movable drives (Diagnostic Biochips; *N* = 2). For fixed arrays, the initial insertion depth was 3.8–4.5 mm below the brain surface. For movable electrodes, the initial insertion depth was 2.6 mm, and electrodes were advanced in 25–50 µm increments after each session until reaching a depth of 4.5 mm. Neural activity was recorded using a multichannel amplifier (Blackrock Neurotech) with a sampling rate of 30 kHz. The number of units recorded from each rat is listed in Extended Data Table 2-1.

### Neural data analysis

Spike sorting was conducted using either Wave_Clus (Chaure et al., 2018) or Kilosort (Pachitariu et al., 2016, 2023; versions 2.5 and 3), assisted by custom MATLAB routines for manual curation. Units were classified as single units if they exhibited consistent spike waveforms, a clear refractory period, and <0.5% of spikes within the 0–3 ms range of the interspike interval histogram. Spike times were recorded at a resolution of 1 ms.

Behavioral event markers were simultaneously recorded with neural data, allowing for alignment between spike times and behavioral events. In the SRT task, the stimulus onset was fixed (either 750 ms or 1,500 ms after the lever press), but RT and the duration from lever release to subsequent actions varied across trials (Fig. 2). Traditional analysis methods align spikes to specific events, generating perievent time histograms (PETHs) for events like lever press, release, and port poke individually. To account for variability across trials, a time-warping procedure was applied to create a single PETH that aligns all events.

For this procedure, the median response time for a variable (e.g., release time) was used as the target time for warping. Spike trains from individual trials were first converted into spike density functions (SDFs) by convolving the spike times with a Gaussian kernel (*σ* = 20 ms). The SDFs were then warped by matching event timings between the current trial and the target time. Let $t=t_{1},t_{2},\ldots ,t_{n}$ be the time of the SDF(t) of the current trial, and let the target time be $t=t_{1}^{\prime },t_{2}^{\prime },\ldots ,t_{k}^{\prime }$. The scaling factor $s=((t_{k}^{\prime }-t_{1}^{\prime })/(t_{n}-t_{1}))\;$was applied to warp the SDF, yielding $\mathrm{SD}F_{\mathrm{warp}}(t),\;t=t_{1}^{\prime },\ldots ,t_{k}^{\prime }$ as follows:

$$\mathrm{SD}F_{\mathrm{warp}}(t_{i}^{\prime })=(\lceil i\cdot s\rceil -i\cdot s)\cdot \mathrm{SDF}(\left\lfloor i\cdot s\right\rfloor )+(i\cdot s-\lfloor i\cdot s\rfloor )\cdot \mathrm{SDF}(\left\lceil i\cdot s\right\rceil ).$$

Here, ⌈⋅⌉ and ⌊⋅⌋ are ceiling and flooring functions, respectively. Following mean subtraction, $\mathrm{SD}F_{\mathrm{warp}}(t)\;$ was normalized to the *z*-score (Fig. 3*A*). Data from correct trials from the long FP (1,500 ms) were used for ranking unit based on their peak response timing. The ranking was then applied to align warped PETHs for the short FP (750 ms).

To visualize population activity in low-dimensional space, we constructed a matrix consisting of all warped PETHs (*z*-scored SDFs) for MO or DLS units separately. Each column corresponded to a unit and each row to a time point. Principal component analysis (PCA) was performed on this matrix using MATLAB's pca function, with the projections of population activity onto the principal components representing population dynamics that captured the most variance in the data.

### Histological analysis

After the behavioral experiments were completed, the rats were deeply anesthetized with 5% isoflurane and transcardially perfused with chilled PBS, followed by 200 ml of 4% paraformaldehyde (PFA) in PBS. The brains were extracted and fixed in PFA for an additional 24 h before being transferred to PBS. Free-floating sections (70–80 µm thick) were prepared using a microtome (Leica Biosystems). These sections were mounted and stained with cresyl violet (Nissl stain; Paul et al., 2008) to quantify the lesioned area. Slices were imaged using a microscope slide scanner (OLYMPUS VS120) and aligned with corresponding coronal sections from the standard rat brain atlas (Paxinos and Watson, 2014) using a semiautomatic procedure in ImageJ (BigWarp; Bogovic et al., 2016). For rats that received spinal injections of retrograde tracers, every other section was mounted on separate slides using a mounting medium mixed with DAPI.

Lesions in the striatum were associated with either increased gliosis or neuronal loss, resulting in a corresponding increase or decrease in the Nissl signal. In a subset of sections, additional staining was performed to further verify the lesioned area. Using standard immunofluorescence staining techniques, sections from lesioned rats were stained with primary antibodies against Foxp1 (ab16645, Abcam) to visualize medium spiny neurons in the striatum. Representative examples are shown in Figure 4*E*.

### Statistics

Statistical analysis was conducted using MATLAB's Statistics and Machine Learning Toolbox. The statistical methods and results for each figure are detailed in the figure legend, and statistics are reported in extended tables. Data from all subjects are generally presented as violin plots, illustrating the empirical probability density function. Where space permitted, the median, interquartile range, and confidence intervals were also indicated. A two-tailed permutation test (Wasserman, 2004) was used when the sample size was limited (<30). For comparisons involving lesion effects and recovery, repeated-measure ANOVA followed by post hoc multiple comparisons [Tukey's honestly significant difference (HSD)] was applied to analyze lesion types and lesion time points (Tables 2, 3).

**Table 2.** Summary of repeated measures ANOVA examining the effect of different lesions

Factors	RT		Premature ratio		Correct ratio	
	*F* value	*p* value	*F* value	*p* value	*F* value	*p* value
Unilateral MO lesions						
FP	*F*_(2,30)_ = 3.394	0.0469	*F*_(2,30)_ = 72.400	<10^−4^	*F*_(2,30)_ = 43.248	<10^−4^
Cond.	*F*_(2,30)_ = 21.775	<10^−4^	*F*_(2,30)_ = 3.945	0.0301	*F*_(2,30)_ = 36.977	<10^−4^
FP x Cond.	*F*_(4,60)_ = 0.998	0.4159	*F*_(4,60)_ = 1.184	0.3270	*F*_(4,60)_ = 2.392	0.0605
Bilateral MO lesions						
FP	*F*_(2,20)_ = 0.759	0.4813	*F*_(2,20)_ = 86.277	<10^−4^	*F*_(2,20)_ = 25.902	<10^−4^
Cond.	*F*_(2,20)_ = 18.764	<10^−4^	*F*_(2,20)_ = 5.820	0.0102	*F*_(2,20)_ = 20.841	<10^−4^
FP x Cond.	*F*_(4,40)_ = 1.175	0.3363	*F*_(4,40)_ = 1.622	0.1875	*F*_(4,40)_ = 0.191	0.9418
Unilateral DLS lesions						
FP	*F*_(2,38)_ = 0.172	0.8430	*F*_(2,38)_ = 49.447	<10^−4^	*F*_(2,38)_ = 39.404	<10^−4^
Cond.	*F*_(2,38)_ = 7.903	0.0014	*F*_(2,38)_ = 17.793	<10^−4^	*F*_(2,38)_ = 35.489	<10^−4^
FP x Cond.	*F*_(4,76)_ = 0.700	0.5940	*F*_(4,76)_ = 2.933	0.0260	*F*_(4,76)_ = 1.181	0.3258
Bilateral DLS lesions						
FP	*F*_(2,48)_ = 3.264	0.0469	*F*_(2,48)_ = 141.093	<10^−4^	*F*_(2,48)_ = 90.197	<10^−4^
Cond.	*F*_(2,48)_ = 10.897	0.0001	*F*_(2,48)_ = 22.393	<10^−4^	*F*_(2,48)_ = 57.090	<10^−4^
FP x Cond.	*F*_(4,96)_ = 2.873	0.0269	*F*_(4,96)_ = 11.276	<10^−4^	*F*_(4,96)_ = 6.490	0.0001

FP, 0.5, 1.0, 1.5 s; condition (Cond.): prelesion (final session), postlesion (first session), postlesion (final session).

**Table 3.** Summary of post hoc multiple comparisons using Tukey's HSD test for the effects of lesions on behavioral variables across time points

FP (s)	Conditions		RT			Premature ratio			Correct ratio		
	1	2	Diff. (2-1; seconds)	SE	*p* value	Diff. (2-1; %)	SE	*p* value	Diff. (2-1; %)	SE	*p* value
Unilateral MO lesions											
0.5	Pre	Post1	0.096	0.016	<10^−4^	8.05	1.77	0.0011	−24.01	2.24	<10^−4^
	Pre	Post2	0.041	0.017	0.0744	0.39	1.17	0.9409	−2.11	2.35	0.6497
1	Pre	Post1	0.085	0.014	<10^−4^	7.91	3.16	0.0601	−17.76	2.53	<10^−4^
	Pre	Post2	0.037	0.014	0.0399	−0.35	2.18	0.9863	−3.41	1.20	0.0309
1.5	Pre	Post1	0.078	0.015	0.0002	3.20	5.05	0.8042	−13.83	5.32	0.0497
	Pre	Post2	0.048	0.017	0.0262	−1.19	4.12	0.9554	−1.76	3.32	0.8580
Bilateral MO lesions											
0.5	Pre	Post1	0.123	0.030	0.0049	8.47	3.85	0.1195	−29.31	6.82	0.0041
	Pre	Post2	0.045	0.016	0.0429	−0.32	0.74	0.9034	−3.35	3.14	0.5551
1	Pre	Post1	0.149	0.035	0.0047	12.62	4.14	0.0302	−29.11	7.14	0.0057
	Pre	Post2	0.045	0.013	0.0140	−0.45	1.54	0.9547	−5.81	3.15	0.2057
1.5	Pre	Post1	0.172	0.031	0.0006	7.93	4.62	0.2468	−30.47	5.25	0.0005
	Pre	Post2	0.069	0.012	0.0006	−1.37	2.47	0.8472	−3.34	3.08	0.5441
Unilateral DLS lesions											
0.5	Pre	Post1	0.051	0.017	0.0183	6.84	1.29	0.0001	−17.44	3.39	0.0002
	Pre	Post2	0.002	0.010	0.9845	1.26	0.60	0.1195	−1.99	2.37	0.6845
1	Pre	Post1	0.034	0.015	0.0909	10.41	2.11	0.0003	−21.60	3.43	<10^−4^
	Pre	Post2	−0.004	0.014	0.9529	2.69	1.22	0.0948	−3.65	1.48	0.0584
1.5	Pre	Post1	0.039	0.017	0.0866	12.83	2.33	<10^−4^	−19.28	2.52	<10^−4^
	Pre	Post2	−0.012	0.012	0.5568	6.54	2.70	0.0636	−6.18	2.67	0.0776
Bilateral DLS lesions											
0.5	Pre	Post1	0.101	0.022	0.0004	8.10	1.49	<10^−4^	−21.84	3.60	<10^−4^
	Pre	Post2	0.046	0.031	0.3289	5.97	1.52	0.0018	−7.78	3.69	0.1090
1	Pre	Post1	0.081	0.018	0.0004	17.02	2.92	<10^−4^	−28.63	2.91	<10^−4^
	Pre	Post2	0.023	0.020	0.5062	15.30	2.57	<10^−4^	−19.43	2.71	<10^−4^
1.5	Pre	Post1	0.123	0.024	0.0001	18.59	4.06	0.0003	−32.41	3.16	<10^−4^
	Pre	Post2	0.018	0.021	0.6692	23.26	4.17	<10^−4^	−24.36	3.76	<10^−4^

Conditions: Prelesion (final session), postlesion first session (Post1), postlesion final session (Post2).

## Results

### Rats performing an SRT task

**Figure 1.**
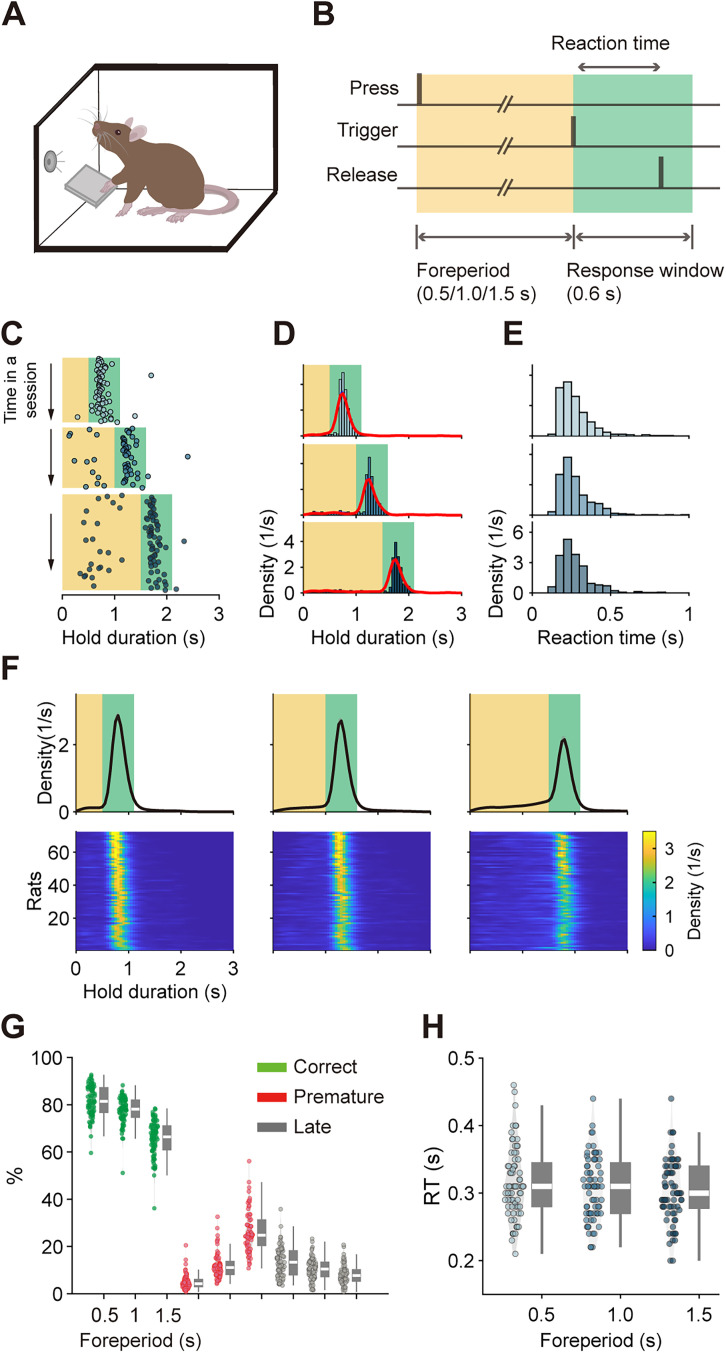
Rats perform an SRT task. ***A***, Cartoon schematic illustrating a rat performing a lever-press SRT task in an operant chamber. ***B***, Task structure. Rats initiated each trial by pressing a lever and held it for a variable FP until a tone appeared. Lever releases before the tone (premature error) or after the response window (late error) were not rewarded. ***C***, Example performance from a single session of a rat. Each dot represents a response (lever release). Yellow and green shades denote the FP and the response window, respectively. Time zero corresponds to the onset of a lever press. Only responses within the green shade were rewarded. For each FP, responses are arranged sequentially from top to bottom as they occurred during the session. ***D***, Empirical probability density functions of release times from an example rat. The red line represents the kernel density estimate. ***E***, Empirical probability density functions of RTs from an example rat. ***F***, Average probability density functions across all rats for three FPs (top). The colormap below illustrates the probability density functions of each rat with color denoting density values. Each row represents a single rat. ***G***, Behavioral performance across all rats. ***H***, RT distribution across all rats. For analysis on the speeding effect, see Extended Data Figure 1-1.

Following previous studies (Amalric and Koob, 1987; Risterucci et al., 2003; Narayanan et al., 2006; Hardung et al., 2017), rats were trained to perform an SRT task using lever release as the conditioned response (Fig. 1*A*,*B*). In this task, rats self-initiated trials by pressing a lever and holding it down for a variable duration until a trigger stimulus appeared. Releasing the lever within a 600 ms response window illuminated a reward port on the opposite side of the behavior box, and nose-poking the port triggered a reward delivery (4% sucrose). Anticipatory (premature) lever releases (before the stimulus) or late releases (after the response window) were not rewarded. Errors resulted in a variable timeout during which the house light was turned off. After a series of training stages (see Materials and Methods for details), rats reached stable performance levels (overall performance >70%) at the final stage, where the FP was randomly set to short, medium, or long durations (0.5, 1, or 1.5 s). An example of SRT performance from a single session of one rat is shown in Figure 1*C*–*E*, demonstrating the rat's ability to hold the lever and promptly release it upon cue. All rats achieved an asymptotically stable response time distribution. The median correct ratios, along with spans from the 25th to 75th percentiles (in parentheses), were as follows: 81.4% (76.7, 87.2%), 77.9% (74.7, 82.0%), and 66.0% (60.9, 71.0%) for short, medium, and long FP, respectively (*N* = 72). The median RTs were 0.31 (0.28, 0.35), 0.31 (0.27, 0.35), and 0.30 (0.28, 0.34) s for short, medium, and long FPs (Fig. 1*F*–*H*).

10.1523/JNEUROSCI.1820-24.2025.f1-1 Figure 1-1 Speeding effect. ***A,*** Reaction times of 35 rats showed a speeding effect where reaction time decreased with longer FPs. Each dot represents the mean reaction time of a single rat. The red dots represent the median reaction time of all rats. The red lines represent the 25th and 75th percentiles. The whiskers represent the range extending beyond 1.5 times the interquartile range (IQR) above or below the 75th and 25th percentiles. ***B,*** Reaction times of 13 rats showed an anti-speeding effect, where reaction time increased with longer FPs. ***C,*** Reaction times of 24 rats showed no significant dependence on foreperiod. Statistical testing was performed by fitting a linear model to the reaction time data with FP as a predictor **RT ~ 1 + FP**. The *p*-value was extracted from the coefficient estimate for FP. The *p*-values for all 72 rats were collected, and a Benjamini-Hochberg procedure was applied to compute a critical p-value (0.03389), ensuring the false discovery rate was controlled at less than 0.05. The regression coefficient associated with FP was considered significant if the raw *p*-value was less than this critical *p*-value. Download Figure 1-1, TIF file.

Previous studies have noted a “speeding effect”—the longer the subject waits, the faster they respond (Niemi and Näätänen, 1981; Brown and Robbins, 1991; Narayanan et al., 2006), indicating implicit signal anticipation and motor preparation as the stimulus becomes more likely to occur. This effect was observed in around half of the rats (35 out of 72; Extended Data Fig. 1-1).

### Neural dynamics of MO or DLS are similar

**Figure 2.**
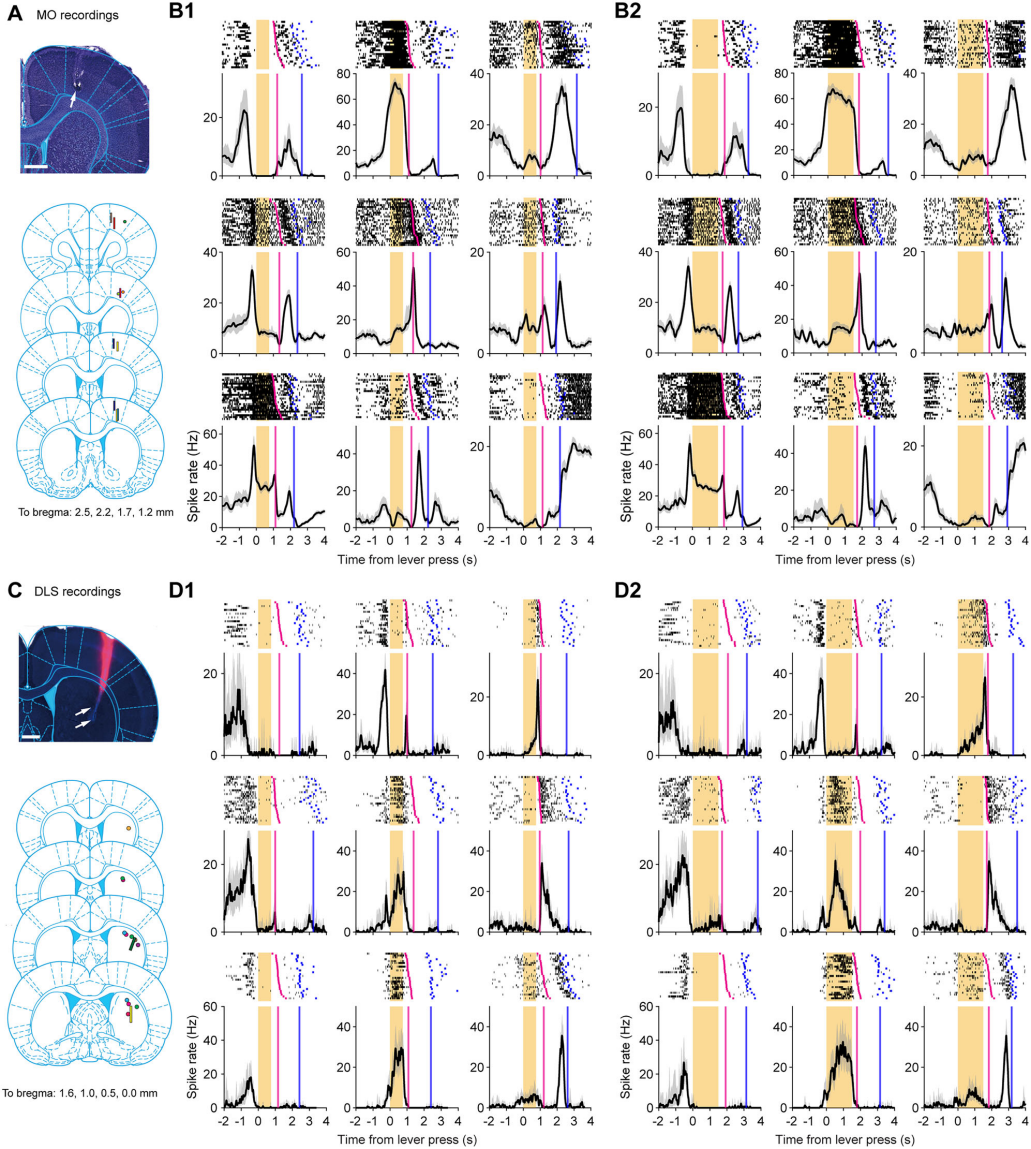
Neural recordings from the MO and DLS during the SRT task. ***A***, Recording sites in the primary MO. Each colored line or circle represents data from an individual rat: lines indicate movable tetrodes or silicon probes, and circles represent fixed microwire arrays. ***B*1**, Spiking activity of nine example units from the MO (FP = 0.75 s). The top panel displays raster plots with spikes in black, ordered by RT (magenta). Poke times are indicated by blue tick marks. The bottom panel shows the peristimulus time histogram (PSTH), time-warped to align with specific events. Shaded regions indicate 95% confidence intervals obtained through bootstrapping. ***B*2**, Same units as in ***B*1** for longer FP trials (FP = 1.5 s). *C*, *D1*, *D2*, Corresponding recording sites and spiking activity from the DLS. See Extended Data Table 2-1 for the number of units recorded per rat.

To explore the neural activity in MO versus DLS during the task, we conducted chronic recordings using multisite electrode arrays when rats performed a modified SRT task with two FPs (0.75 and 1.5 s). Spike trains from sorted units were aligned to key behavioral events—lever press, stimulus delivery, lever release, and reward port approach (Fig. 2). A time-warping procedure was used to normalize trials with different RTs or release-to-poke latencies (see Materials and Methods).

10.1523/JNEUROSCI.1820-24.2025.t2-1 Table 2-1 Number of units recorded from each rat, related to Figure 2. Download Table 2-1, XLSX file.

**Figure 3.**
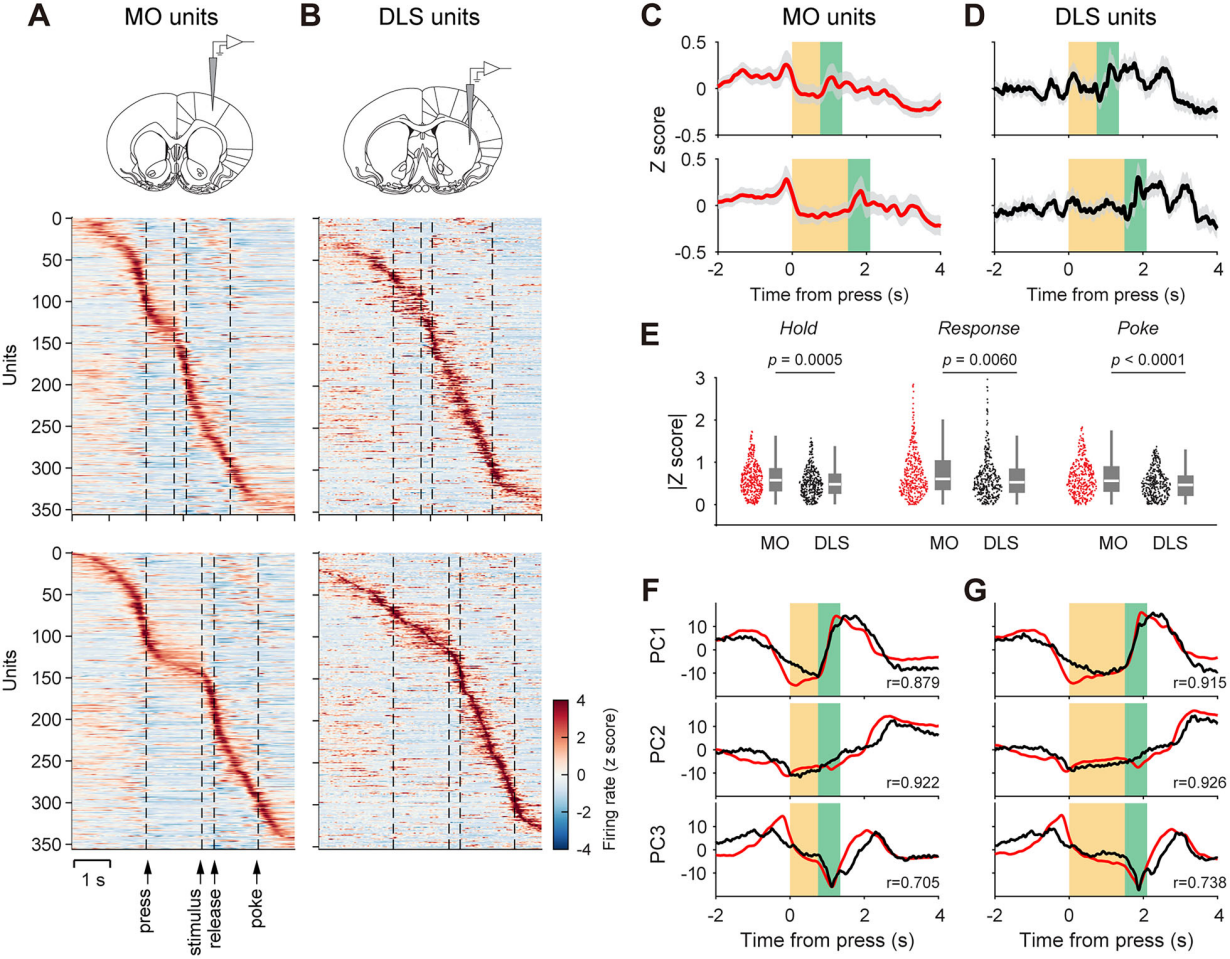
Neural dynamics in the MO and DLS during the SRT task. ***A***, Time-warped PSTHs of spiking activity in MO during the SRT task. The top colormap shows data from trials with a 0.75 s FP, and the bottom colormap shows data from trials with a 1.5 s FP. Neurons are ranked by the timing of their peak firing rate in the 1.5 s FP condition. ***B***, PSTHs for spiking activity in the DLS. ***C***, Average *z*-scored firing rates across all recorded MO units. The top plot represents the 0.75 s FP, and the bottom plot represents the 1.5 s FP. Shaded areas indicate 95% confidence intervals. ***D***, Same as ***C*** for DLS units. ***E***, Average absolute *z*-score of MO and DLS units during three task periods: the FP (Hold), from stimulus presentation to lever release (Response), and from lever release to reward port entry (Poke). Data are presented as scatterplots with box plots (each dot represents a unit). *P* values are from a Wilcoxon rank-sum test. ***F***, Principal component projections of population activity from MO (black) and DLS (red) during the 0.75 s FP, displaying the first three principal components (PC1–PC3). The correlation coefficient (*r*) between MO and DLS population activity is indicated for each component. ***G***, Same as ***F*** but for the 1.5 s FP.

Neurons in both areas were active during behavior, modulated by behavior-relevant sensory or motor events (Jensen et al., 2022). Despite a significantly higher average spike rate in MO neurons than DLS neurons (MO, 9.9 ± 9.6 Hz; *N* = 356; DLS, 6.2 ± 8.2 Hz; *N* = 353; mean ± SD, *t*_(707)_ = 5.65; *p *< 10^−4^; *t* test), the population dynamics between the two regions were similar (Fig. 3*A*). MO and DLS contained subsets of neurons that fired to key phases during the task, such as lever approaching, lever pressing, lever holding, releasing, and reward port approaching (Fig. 3*A*–*D*). Overall, indicated by the absolute average *z*-score computed over three time periods—during the FP, from stimulus to lever release and from lever release to port poking—MO neurons were slightly more modulated than DLS neurons (Fig. 3*E*). PCA revealed comparable population activity dynamics projected to the first three principal axes in both areas (Fig. 3*F*,*G*). The three leading principal components represent 20.6, 17.9, and 11.8% of the total variance in MO units and 16.8, 11.8, and 8.9% in DLS units. The first principal component of both populations showed sustained modulation during the FP, which changed abruptly at the end of FP (Fig. 3*F*,*G*, PC1). Thus, during behavior, MO and DLS neurons exhibit analogous neural dynamics (Park et al., 2025).

### MO and DLS lesions produced dissociable effects on SRT performance

**Figure 4.**
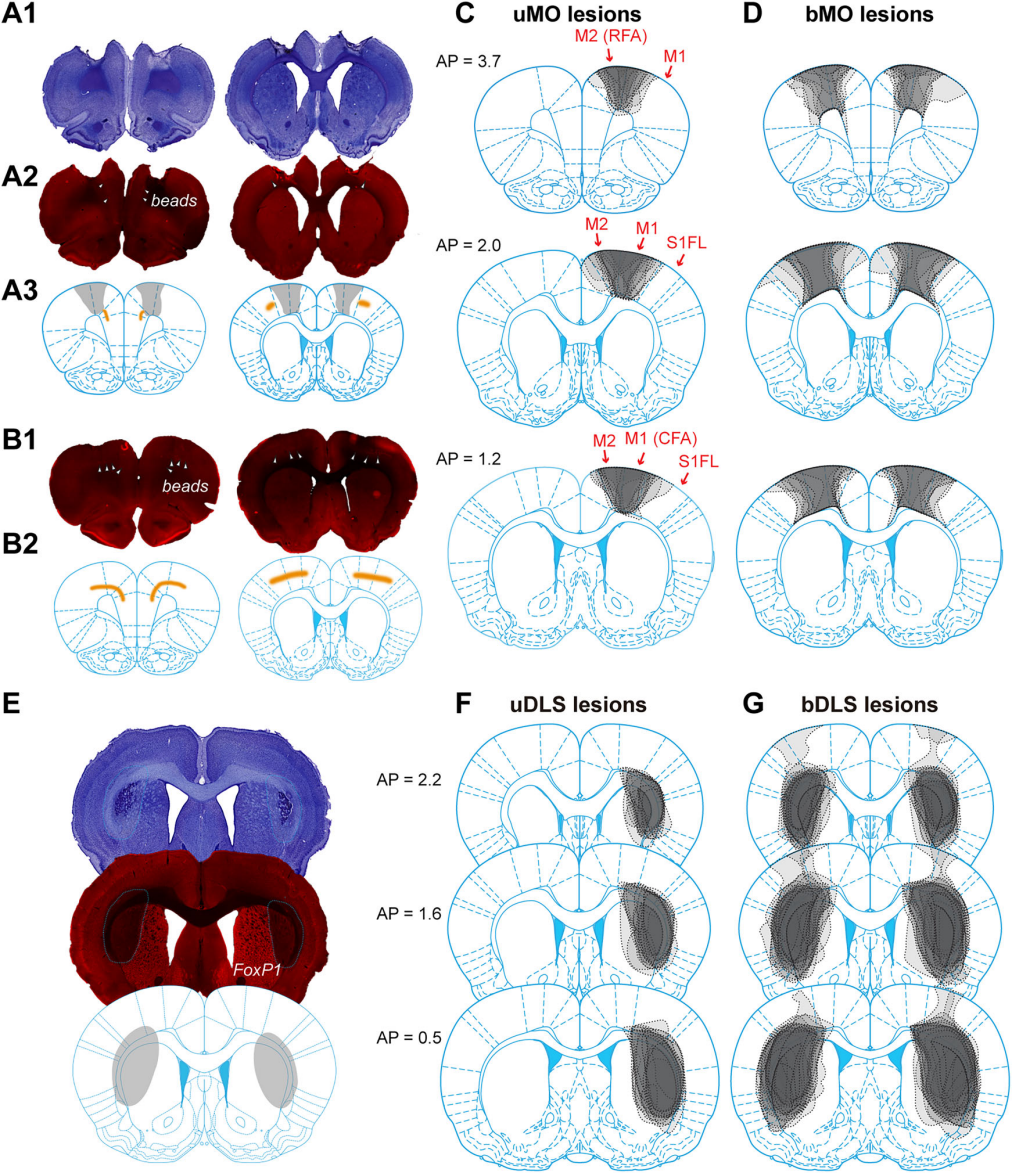
Histology reconstruction of lesioned brains. ***A*1**, Examples of bMO lesions, visualized with Nissl staining. ***A*2**, The same brain slices as in ***A*1**, showing retrogradely transported beads from the spinal cord. ***A*3**, Outlines of the lesioned regions, mapped onto the corresponding brain atlas. The orange-shaded regions indicate the location of the retrogradely transported beads. ***B*1*, B*2**, Brain slices from a rat without MO lesions, displaying beads as a control. ***C***, Lesioned areas in rats with unilateral MO (uMO) lesions, mapped onto three standard coronal brain slices. ***D***, Lesioned areas in rats with bMO lesions, mapped onto three standard coronal brain slices. ***E***, Example of bilateral DLS lesions. Top, Nissl-stained section. Middle, FoxP1 immunofluorescent staining. Bottom, Outlines of the lesioned region mapped onto the standard brain atlas. ***F, G***, Lesioned areas in rats with uDLS and bDLS lesions, respectively, mapped onto standard coronal slices. See Extended Data Table 4-1 for the list of rats in each group.

Although MO and DLS neurons share similarities in their activity during behavior, lesions in these regions may lead to distinct effects on behavioral performance. To investigate this, we injected quinolinic acid into the targeted brain regions to create localized excitotoxic lesions (Fig. 4). Four lesion types were performed: unilateral MO or DLS lesions contralateral to the preferred paw (the paw predominantly used to initiate lever presses) and bilateral lesions targeting either the MO or DLS on both sides of the brain (Fig. 4).

10.1523/JNEUROSCI.1820-24.2025.t4-1 Table 4-1 Rats included in different lesion groups and the number of their pre- and post-lesion training sessions, related to Figure 4. Download Table 4-1, XLSX file.

**Figure 5.**
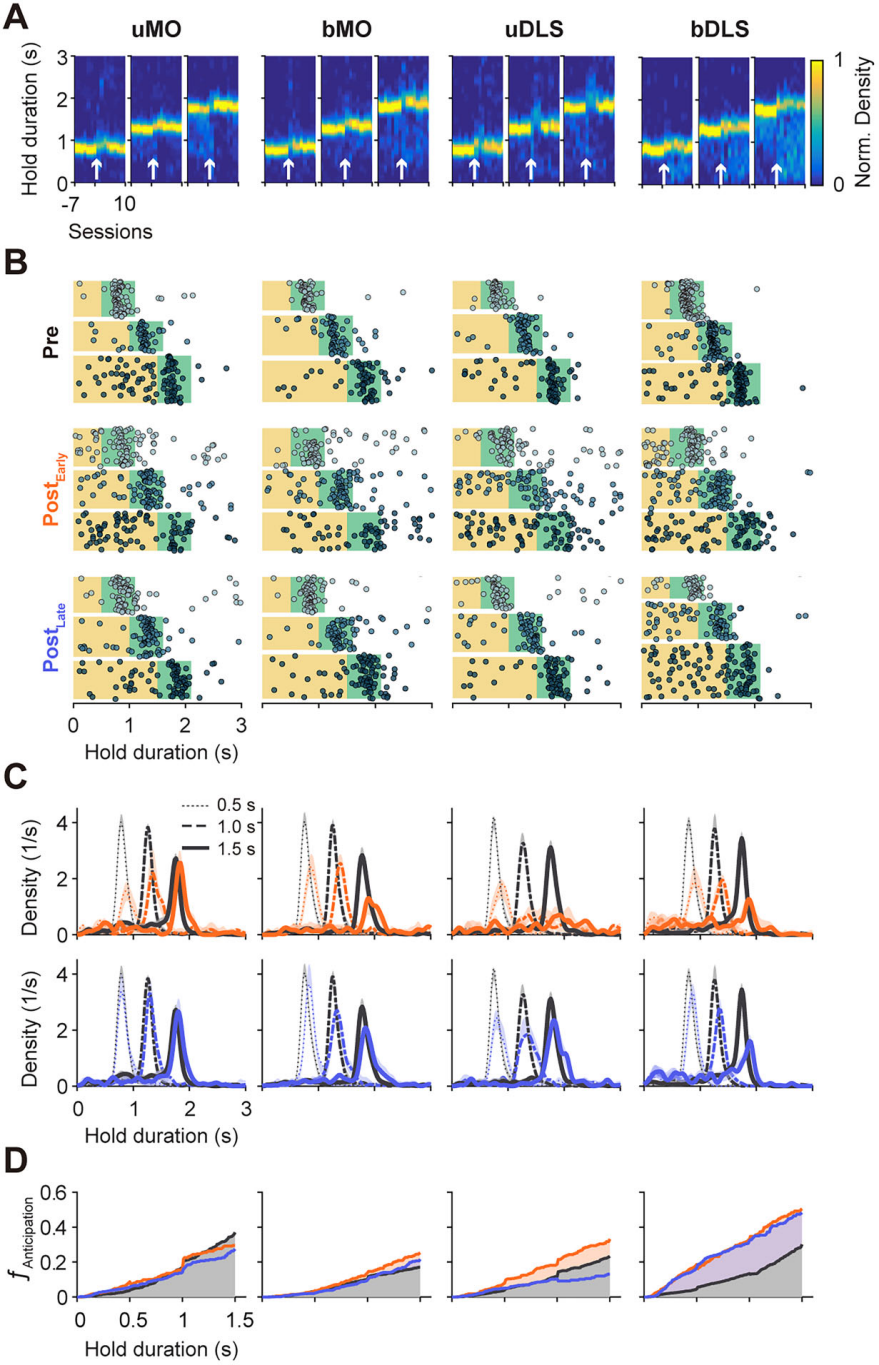
Examples of behavioral performance before and after lesions. ***A***, Probability density functions of responses (normalized to the prelesion condition) across sessions before and after the lesion. Each column in the colormap represents a single session or a concatenation of multiple sessions to meet a minimum trial number of 50. For each lesion type, three colormaps correspond to the three different FPs. ***B***, Example performance from three time points: prelesion (Pre), initial postlesion sessions (Post_Early_), and late postlesion sessions after extensive training (Post_Late_). Responses from Pre and Post_Late_ conditions are from single sessions, with responses arranged from top to bottom according to their occurrence in a session (as shown in Fig. 1*C*). Responses from Post_Early_ condition are concatenated from up to three sessions. ***C***, Probability density functions of hold durations under different conditions. Top panel compares Pre responses with Post_Early_ responses. Bottom panel compares Pre responses with Post_Late_ responses. ***D***, Effects of lesions on the anticipation function.

**Figure 6.**
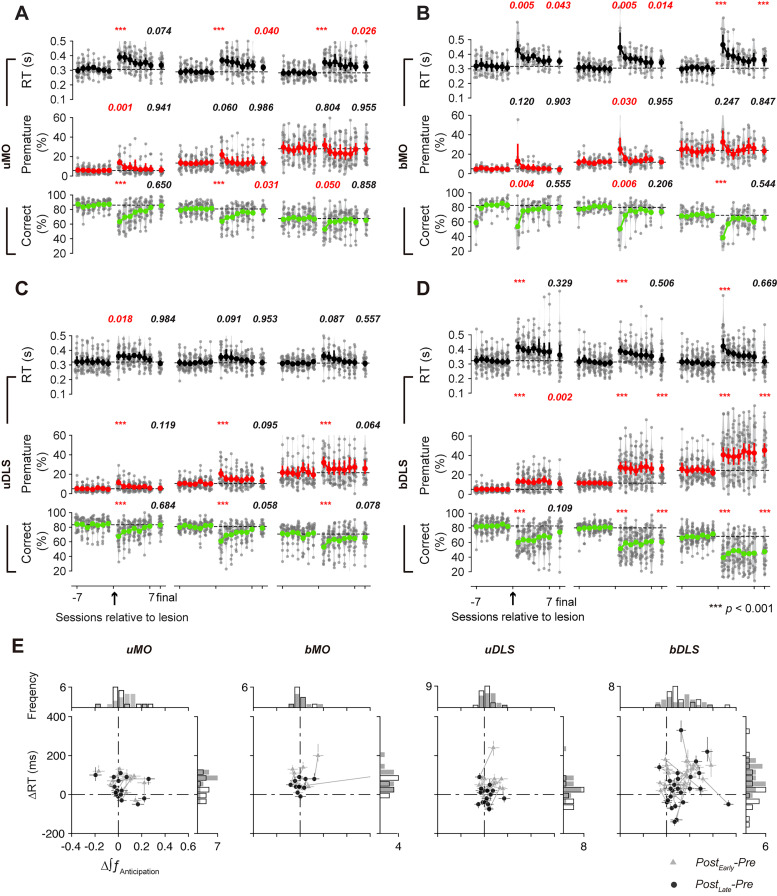
Behavioral deficits and recovery following different types of lesions. ***A–D***, RT, premature response ratio, and correct response ratio across sessions for different lesion types. Each dot represents data from a single rat, with 95% confidence intervals displayed for each session. Results for each lesion type are presented in order from left to right for FP of 0.5, 1.0, and 1.5 s. *P* values are from tests comparing the final prelesion session and the first postlesion session or comparing the final prelesion session and the last postlesion session (repeated-measure ANOVA followed by post hoc Tukey's HSD multiple comparisons). ***E***, Relationship between changes in the integral of the anticipation function and changes in RT during Post_Early_ and Post_Late_ sessions across rats. Error bars represent 95% confidence intervals. Gray lines connect data from the same subjects. Histograms display the distribution of changes in RT or the integral of anticipation function, with gray bars indicating changes in Post_Early_ sessions and white bars indicating changes in Post_Late_ sessions. See Extended Data Table 6-1 for data from all rats.

Both unilateral (uMO) and bilateral (bMO) lesions of the MO caused an initial decline in task performance which recovered over several training sessions (example rat, Fig. 5; all rats, Fig. 6*A*,*B*; uMO, *F*_(2,30) _= 36.977; *p *< 10^−4^; bMO, *F*_(2,20) _= 20.841; *p *< 10^−4^; repeated-measure ANOVA on correct response ratio; Tables 2, 3). A within-subject permutation test (10,000 permutations) was applied to examine significant changes in individual subjects at different time points. Significant differences were identified by comparing raw *p* values to a critical threshold of 0.0336, obtained by applying the Benjamini–Hochberg procedure for controlling the false discovery rate at 0.05. This threshold was derived from 288 permutation tests (Extended Data Table 6-1): 72 rats × 2 tests (Pre vs Post_Early_, Pre vs Post_Late_) for RT (pooled across all FPs) plus 72 × 2 tests for premature responses (see below). Most subjects exhibited increased RTs in early postlesion sessions (uMO, 13 out of 16 rats showed significant increases, and 1 showed a decrease; bMO, 10 out of 11 rats showed increases, and 0 showed a decrease)*.* Across rats, uMO lesions increased RTs by a median of 80 ms (25th–75th percentile span = 55–100 ms; paired permutation test on group data; *p *< 10^−4^; *N* = 16; Fig. 6*A*,*E*), while bMO lesions increased RTs by 60 ms (span, 40–125 ms; *p *< 10^−4^; *N* = 11; Fig. 6*B*,*E*).

10.1523/JNEUROSCI.1820-24.2025.t6-1 Table 6-1 Summary of changes in reaction time and anticipatory responses following lesions across rats, related to Figure 6E. Download Table 6-1, XLSX file.

Changes in premature response were quantified by calculating the difference of the area under the anticipatory functions (Ollman and Billington, 1972) between pre- and postlesion trials (Figs. 5*D*, 6*E*) or by estimating the premature error rate for each FP (Fig. 6*A*,*B*). The effects of lesions on premature responses were variable: some rats showed significant increases (uMO, nine rats; bMO, four rats; Extended Data Table 6-1), while others showed reductions (uMO, two rats; bMO, three rats). Across rats, the changes in premature responses were small (uMO, median, 0.07; span, 0.00–0.11; *p *= 0.059; bMO, median, 0.02; span, −0.04–0.07; *p *= 0.276). Postlesion training (median, 10 sessions) improved RT and reduced premature responses. By the end of postlesion training, RTs remained mildly longer compared with the prelesion levels (uMO, nine rats showed increases, and three showed decreases; median, 20 ms; span, −5–85 ms; *p *= 0.0151; bMO, nine rats showed an increase, and zero showed decreases; median, 40 ms; span, 36–78 ms; *p *= 0.0001). However, the premature responses were no longer significantly different from the prelesion level (uMO, four rats showed increases, and one showed a decrease; median, 0.01; span, −0.02–0.05; *p *= 0.2701; bMO, one rat showed an increase, and one showed a decrease; median, −0.01; span, −0.03–0.03; *p *= 0.982).

Both unilateral (uDLS) or bilateral (bDLS) DLS lesions also impaired behavioral performance (uDLS, *F*_(2,38) _= 35.489; *p *< 10^−4^; bDLS, *F*_(2,48) _= 57.090; *p *< 10^−4^; Fig. 6*C*,*D*; Tables 2, 3). uDLS lesions produced an initial increase in RT (15 out of 20 rats showed significant increases, 1 showed a decrease; median, 38 ms; span, 15–70 ms; *p *< 0.0001; *N* = 20) and a rise in premature responses (11 showed significant increases, 0 showed decreases; median, 0.05; span, 0.00–0.08; *p *= 0.002; Extended Data Table 6-1; Figs. 5, 6*C*,*E*). These deficits recovered with training, with no significant difference in RT (median, 5 ms; span, −40–20 ms; *p *= 0.615; 10 showed significant increases and 9 showed decreases) or premature responses (median, 0.02; span, −0.03–0.04; *p *= 0.158; five showed significant increases; one showed a decrease) compared with prelesion levels.

In contrast, bDLS lesions initially caused a pronounced increase in RT (median, 100 ms; span, 50–143 ms; *p *< 10^−4^; *N* = 25; 22 out of 25 rats showed significant increases, and 0 showed decreases) and premature responses (median, 0.15; span, 0.01–0.21; *p *< 10^−4^; 16 showed significant increases and 2 showed decreases; Extended Data Table 6-1; Figs. 5, 6*D*,*E*). After extended training (median, 20 sessions), the premature responses remained elevated (median, 0.10; span, 0.04–0.20; *p *< 10^−4^; 18 showed significant increases, and 1 showed a decrease). Changes in RT were variable across rats (median, 30 ms; span, −40–95 ms; *p *= 0.077; 14 showed significant increases and 8 showed decreases; Fig. 6*E*; Extended Data Table 6-1).

A three-way ANOVA revealed a significant interaction among lesion types, lesion time points (prelesion, postlesion first session, postlesion last session), and FP for premature response rates (*F*_(12,272) _= 3.865; *p *< 10^−4^), consistent with specific effects of bilateral DLS lesions on premature responses. In contrast, the three-way interaction for RT was only marginally significant (*F*_(12,272) _= 1.6564; *p *= 0.076).

**Figure 7.**
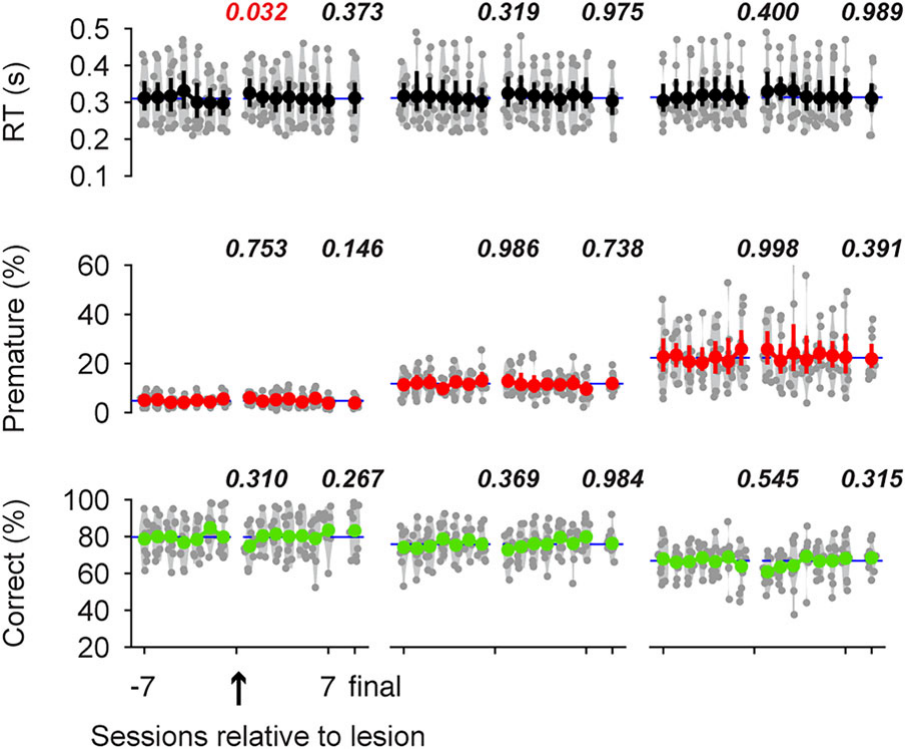
Sham surgery produced no systematic deficits in behavior. Figure layout is the same as Figure 6*A*. *P* values are from tests comparing the final pre-sham session and the first post-sham session or comparing the final pre-sham session and the final post-sham session (repeated-measure ANOVA followed by post hoc Tukey's HSD multiple comparisons).

Finally, sham operations caused no significant impairment in the premature response rate (*F*_(2,20)_ = 2.039; *p *= 0.156) or RT (*F*_(2,20)_ = 2.679; *p *= 0.093; *N* = 11; Fig. 7), suggesting that the experimental procedure and the postsurgery recovery period (7–10 d) did not adversely affect behavioral performance. There was a slight trend toward improved behavioral performance over extended training sessions following the sham operation (*F*_(2,20)_ = 12.5065; *p *= 0.0003). Notably, significant improvements in correct responses were observed between the first and final post-sham sessions for the shortest FP (8.4 ± 2.7%; mean ± SEM; *p *= 0.026) and a marginally significant improvement of 7.6 ± 2.8% (*p *= 0.053) for the longest FP.

### Bilateral DLS lesions shifted the timing of self-timed responses

**Figure 8.**
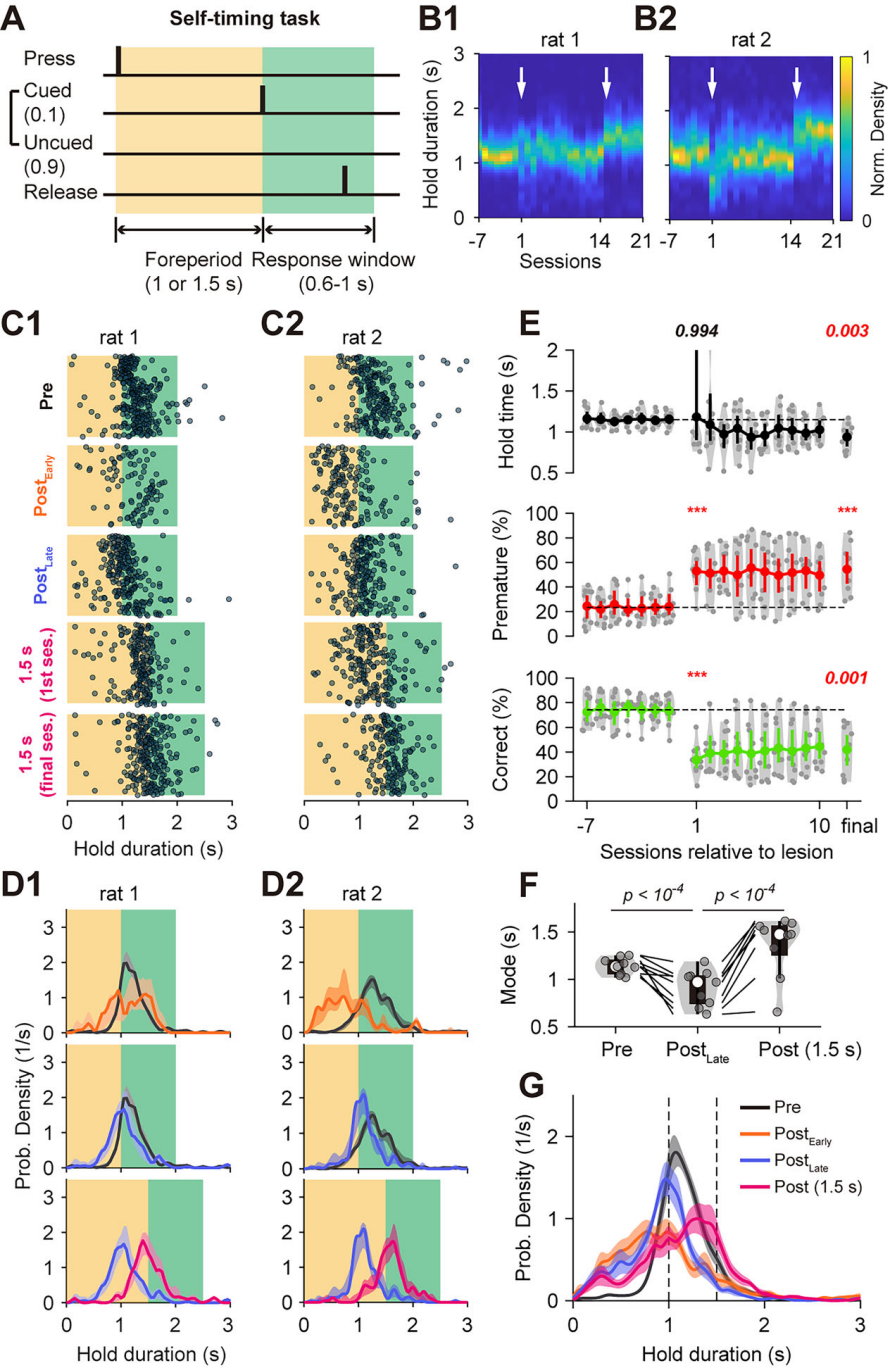
Behavioral deficits in self-timing task following bilateral DLS lesions ***A***, Structure of the self-timing task. “Cued” (10%) and “Uncued” (90%) trials are interleaved throughout the session. ***B*1, 2**, Probability density functions (normalized to prelesion) of two example rats performing the self-timing task. Data are shown before and after bilateral DLS lesions (first arrow) and after switching to a longer FP (1.5 s; second arrow). ***C*1,2**, Example responses from the final prelesion session, early and final postlesion sessions, the first and final 1.5 s FP sessions. Responses are arranged from top to bottom according to their occurrence in a session. ***D*1, 2**, Probability density functions comparing Pre (black) and Post_Early_ (orange) responses (first row), Pre and Post_Late_ (blue) responses (second row), and responses from the final 1 s task (blue) versus the final 1.5 s task (magenta; third row). ***E***, Session-by-session changes in median hold duration, premature response ratio, and correct response ratio following DLS lesions. Each dot represents data from a single rat, with the mean and 95% confidence intervals displayed for each session. *P* values indicate comparisons between the final prelesion and the first postlesion sessions or between the final prelesion and the final postlesion sessions (repeated-measure ANOVA followed by post hoc multiple comparisons). ***F***, Mode of the response distribution of all rats before the lesion, in the final session after lesion, and in the final session after switching to 1.5 s FP. Each dot represents a single rat. Statistical tests were performed using a paired permutation tests with 10,000 permutations. ***G***, Average probability density functions across different conditions, with shaded areas representing SEM.

Thus, in the SRT task, bilateral DLS lesions permanently impaired the rats’ ability to postpone a response—that is, to wait. We next asked whether DLS lesions affect behavior similarly when the action timing is internally determined. To address this, we trained rats on a self-timing task that required them to press and hold a lever for a fixed FP of 1 s (Fig. 8*A*). In most trials (90%), no signal was provided at the end of the FP, requiring the rats to release the lever based on their own motor timing. Over time, rats developed a consistent distribution of release times centered around the end of the FP, suggesting a strong tendency for time anticipation (two example rats: Fig. 8*B–D*; grand average across all rats: Fig. 8*G*, black curve).

To estimate the mode of the response distribution, we fitted a Gaussian model to the response times using MATLAB's *fit* function (based on Levenberg–Marquardt algorithm) and took the center of the Gaussian distribution as the mode. The mode of the self-timed responses occurred slightly after the FP (median, 1.14 s; min–max range, 1.02–1.25 s; nine rats; Fig. 8*F*).

Following bilateral DLS lesions, rats immediately showed a substantial decline in correct response ratios (*F*_(2,16) _= 26.069; *p *< 10^−4^; repeated-measure ANOVA; examples, Fig. 8*B*,*C*; group correct ratio, prelesion final session, 73.8 ± 4.5%; postlesion first session, 33.6 ± 4.5%; Δ = −40.2 ± 5.1%; mean ± SEM; Fig. 8*E*). Premature responses (i.e., releases before 1 s) also rose markedly (*F*_(2,16) _= 16.334; *p *< 10^−4^; prelesion, 23.9 ± 4.4%; postlesion, 53.1 ± 5.2%; Δ = 29.1 ± 4.6%; Fig. 8*E*). Examination of postlesion performance revealed that lesioned rats did not show significant improvement with training (*F*_(10,80) _= 0.3692; *p *= 0.959; repeated-measure ANOVA on premature ratio with 9 subjects and 11 postlesion sessions). By the final postlesion session, the premature response rate remained elevated at 54.5 ± 6.7% (Δ = 30.6 ± 5.1% relative to prelesion; number of postlesion sessions, median, 18; min–max range, 14–23), and the correct response rate remained strongly reduced at 42.0 ± 6.8% (Δ = −31.8 ± 5.7%).

Across all rats, the mode of responses shifted leftward following lesions (median, 0.97 s; min–max range, 0.63–1.19 s; Fig. 8*F*,*G*), in line with the persistent tendency in premature responding. Although behavioral performance did not improve further in the 1 s task, when the FP was extended to 1.5 s, all lesioned rats adaptively shifted their response times to the right (median of mode, 1.48 s; min––max range, 0.66–1.62 s; Fig. 8*B*–*D*,*F*,*G*; compare blue and magenta lines). This suggests that the rats did not develop a lesion-induced motor impairment in holding the lever for an extended period.

### Bilateral DLS lesions slowed down the body and paw movements

**Figure 9.**
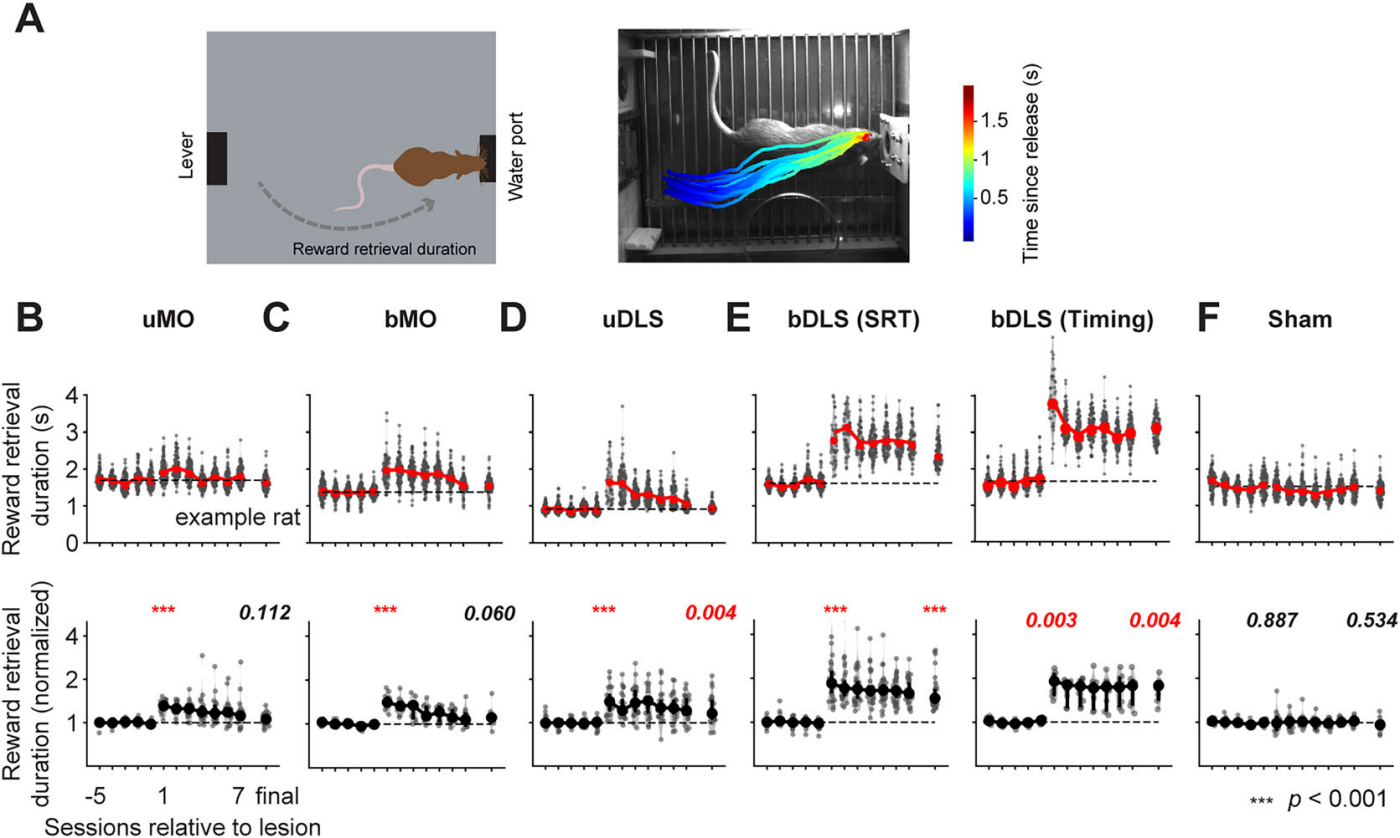
Effects of lesions on gross movement. ***A***, Left, Schematic illustrating the movement trajectory of a rat from lever release to poking at the water port. Right, Example movement trajectories, color-coded to indicate time elapsed from lever release. ***B***, Top, Reward retrieval duration from lever release to water port poking for an example rat. Each violin plot represents the distribution of durations for a single session, with each data point representing a single trial. Data from five consecutive prelesion sessions and seven consecutive postlesion sessions (after unilateral MO lesion) are shown, with an additional final session displayed at the end. Bottom, Reward retrieval duration from all rats in the uMO lesion group. For each rat, the duration is normalized to the average prelesion value. *P* values indicate post hoc multiple comparisons between the final prelesion session and the first postlesion session or between the final prelesion session and the final postlesion sessions following repeated-measure ANOVA (uMO, *F*_(2,30) _= 36.672; *p *< 10^−4^). ***C–E***, Reward retrieval durations for rats with other types of lesions (bMO, *F*_(2,20) _= 45.583; uDLS, *F*_(2,38) _= 16.999; bDLS, *F*_(2,46) _= 28.200 for SRT task and *F*_(2,16) _= 20.029 for self-timing task; *p *< 10^−4^ for all lesion types). Data from the SRT and self-timing tasks are shown separately for bilateral DLS lesions. ***F***, Reward retrieval durations for rats with sham operations (*F*_(2,20) _= 0.774; *p *= 0.475).

Some previous studies suggest that the basal ganglia, including DLS, is critical for enhancing movement vigor, for example, movement velocity and acceleration (Turner and Desmurget, 2010; Dudman and Krakauer, 2016; Jurado-Parras et al., 2020; Morvan et al., 2024). In our experiments, each correct release triggered the reward port light, prompting the rats to turn and run to collect their reward (Fig. 9*A*). The duration between a correct lever release and the subsequent poke to the reward port was defined as the “reward retrieval duration,” reflecting the rats' gross body movement speed, a measure of motor vigor. The reward retrieval duration increased acutely following all types of lesions (Fig. 9*B*–*E*; post/pre, uMO, 132 ± 3%; bMO, 144 ± 6%; uDLS, 144 ± 7%; bDLS, 522 ± 319%; mean ± SEM). However, bilateral DLS lesions caused a particularly severe and persistent impairment, with latencies still at 174 ± 13% of prelesion levels even after extensive training (*p *< 10^−4^). In contrast, other lesion types, in particular, MO lesions, had weaker lasting effects (uMO, 108 ± 3%; *p *= 0.1121; bMO, 114 ± 6%; *p *= 0.0605; uDLS, 129 ± 7%; *p *= 0.0036). A two-way ANOVA revealed a significant interaction between lesion types and lesion time points (*F*_(6,134) _= 6.5719; *p *< 10^−4^). The effect of bilateral DLS lesions on reward retrieval duration was also observed in the self-timing task (Fig. 9*E*, right). Thus, DLS lesions, in particular bilateral DLS lesions, severely slowed down whole-body movement. Sham operations had no significant effect on reward retrieval duration (Fig. 9*F*).

**Figure 10.**
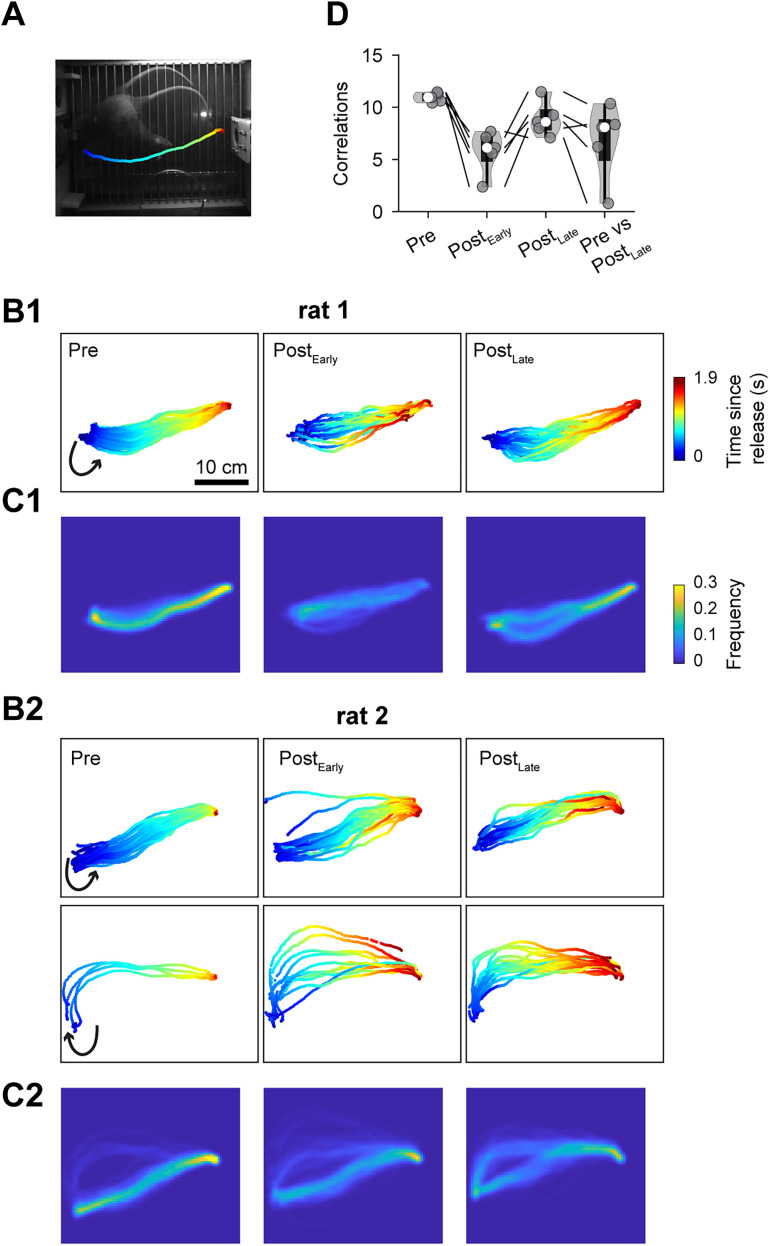
Effects of bilateral DLS lesions on locomotor movement trajectory. ***A***, Image of a rat making a counterclockwise turn after releasing the lever and heading toward the water port (located on the right of the image). ***B***1, Example movement trajectories of a rat at three different time points relative to the lesion: the final session before the lesion (Pre), the first session after the lesion (Post_Early_), and the final postlesion session (Post_Late_). The trajectory is color-coded to indicate the time elapsed since lever release. ***C*1**, Averages of movement trajectories mapped to a 2D spatial grid. ***B*2**, ***C*2**, Examples from a second rat, showing two distinct movement patterns: one counterclockwise and one clockwise. ***D***, Averaged correlation values for movement trajectories at the same time points relative to the lesion or between two time points (Pre ∼ Post_Late_). Lines connect values from the same rat across different time points. See Movie 1.

To examine changes in locomotor movement patterns, we tracked the movement trajectories of five rats before and after bilateral DLS lesions (Fig. 10*A*). The movement trajectory, from lever release to port poking, was imaged by tracking the right ear (Fig. 10*B*; Mathis et al., 2018). These trajectories were then projected onto an 800 × 700 (width × height) 2D grid, where each grid unit (0.4 × 0.4 cm) was marked as 1 if crossed and 0 otherwise, generating a binary matrix. The averaged matrix provides an overall representation of the animal's movement patterns (Fig. 10*C*). In addition, the correlation between any two trajectories was calculated as the dot product of the projections, with highly similar trajectories yielding a high positive correlation score, whereas trajectories with low similarity produced a score closer to zero. We computed correlations for all possible pairs of trajectories within a condition [prelesion (Pre), postlesion early sessions (Post_Early_), and postlesion final session (Post_Late_)] or between two conditions (Pre vs Post_Late_).The movement trajectories remained consistent across different time points (Fig. 10*B*,*C*). That is, lesioned rats did not develop drastically abnormal movement patterns due to locomotion impairment (Movie 1). However, movement trajectory correlations within the same time points after the lesion showed a significant reduction (*p *< 0.01 for each rat). Specifically, correlations were 11.0 ± 0.2 in Pre and 8.9 ± 0.7 in Post_Late_, corresponding to a 19 ± 6% reduction (mean ± SEM, *N* = 5), indicating an increase in movement variability. Additionally, the correlation between Pre and Post_Late_ sessions was 6.8 ± 1.6, which was reduced in each rat compared to within-session correlations in Pre (39 ± 15% reduction; Fig. 10*D*), suggesting a slight change in movement trajectory after the lesion.

**Figure 11.**
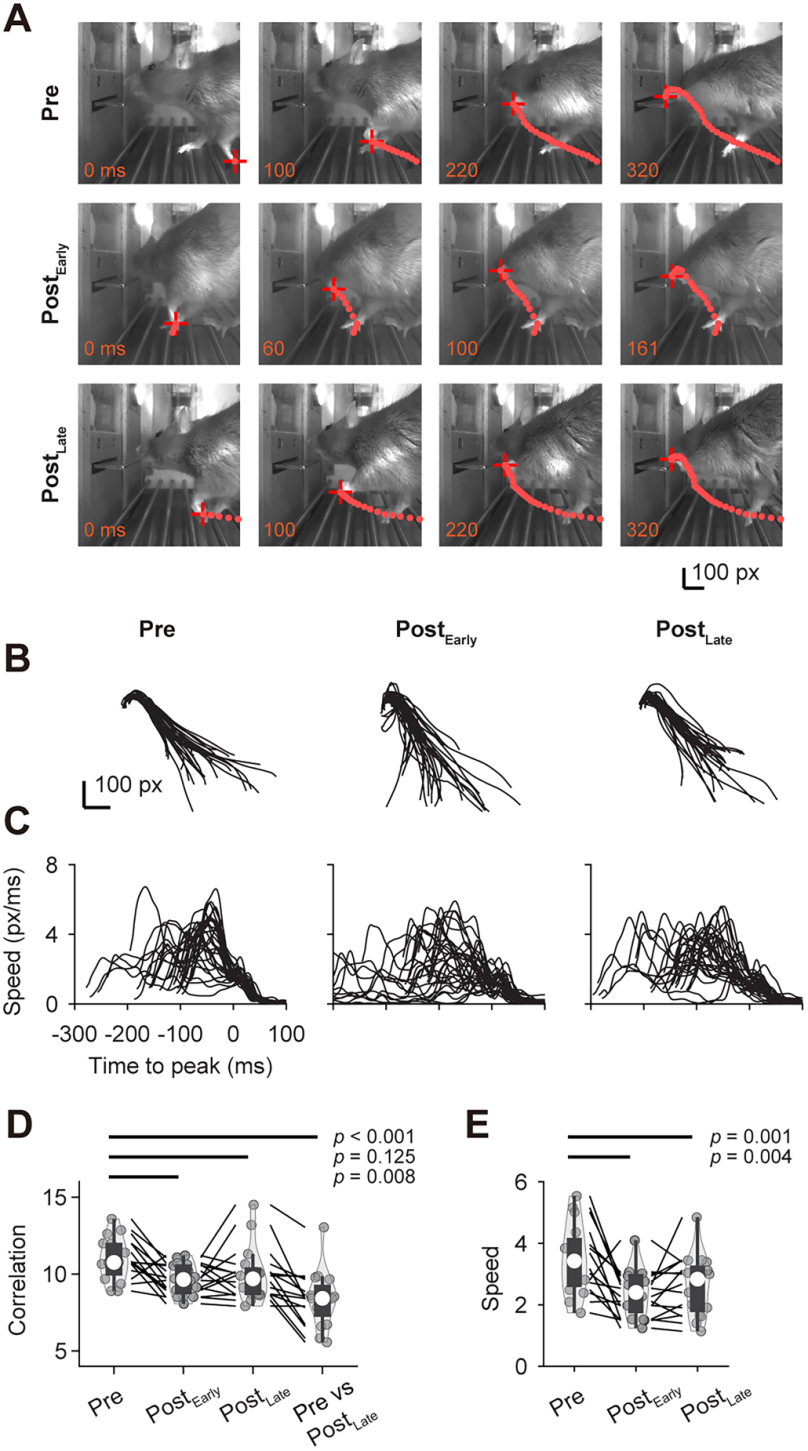
Effects of bilateral DLS lesions on paw trajectories and movement speed. ***A***, Example paw trajectory from a single trial before and after bilateral DLS lesions. Each dot represents the paw's location, with a 10 ms interval between successive dots. Numbers in the frame indicate the relative time. Post_Early_ and Post_Late_ indicate trials in initial sessions after the lesion and after extended training, respectively. ***B***, Example paw trajectories from a single rat, before and after bilateral DLS lesions. ***C***, Speed as a function of time for exemplar paw trajectories in ***B***, before and after bilateral DLS. ***D***, Paw trajectory correlations for rats (*N* = 16) with bilateral DLS lesions (*F*_(3,45) _= 14.464; *p *< 10^−4^). ***E***, Paw lifting speed for rats with bilateral DLS lesions (*F*_(2,30) _= 13.762; *p *< 10^−4^). Statistical tests were performed by repeated-measure ANOVA followed by *post hoc* multiple comparisons. See Movies 2 and 3.

Furthermore, DLS lesions may lead to significant motor deficits in paw movement, potentially preventing subjects from reaching for the lever and thus completing the task (Dhawale et al., 2021). To examine this, we tracked paw movement trajectory before and after lesions (Fig. 11*A,B*). These trajectories were also mapped onto a 2D grid. Bilateral DLS lesions initially reduced the correlation of individual lever-reaching movements (from 11.0 ± 0.4 to 9.6 ± 0.3; 11.1 ± 3.0% reduction; mean ± SEM; *N* = 16) and decreased movement speed (from 3.5 ± 0.3 to 2.4 ± 0.2 pixels/ms; 27.2 ± 6.8% reduction) in early postlesion sessions (Fig. 11*B*–*E*). With postlesion practice, both the correlation (10.0 ± 0.5; 8.6 ± 3.6% reduction) and speed (2.6 ± 0.2 pixels/ms; 23.4 ± 4.8% reduction) showed improvement. The correlation between pre- and postlesion movement trajectories (8.4 ± 0.5; 22.8 ± 4.2% reduction) remained much lower than within-condition correlations, suggesting that the lesion induced a long-lasting change in movement patterns, indicating behavioral compensation (e.g., rats moved closer to the lever before initiating a press). Despite the reduced trajectory correlation and slower paw speed, bDLS lesions did not abolish the rats' ability to perform the task, as they could still reach for, press, and hold the lever (Movies 2 and 3).

**Movie 1.** Example of a rat retrieving reward after lever release before and after bilateral DLS lesions. The video is slowed down by 3.33-fold. [View online]

**Movie 2.** Example of a rat performing the SRT task before and after bilateral DLS lesions. The video is slowed down by twofold. [View online]

**Movie 3.** Example of a rat performing the self-timing task before and after bilateral DLS lesions. The video is slowed down by twofold. [View online]

## Discussion

To investigate the neural substrates underlying the decision between waiting and responding, we performed excitotoxic lesions in the MO or the DLS in an SRT task, which involved balancing waiting (Bari and Robbins, 2013) and rapid reaction to a stimulus (Laubach et al., 2015; Narayanan and Laubach, 2017). Both MO and DLS contained neurons relevant to the task, including those that fired spikes when the subjects waited for the stimulus. Lesions in either region—unilaterally or bilaterally—initially caused slower response times and increased premature response rates. However, only rats with bilateral DLS lesions developed a persistent deficit in waiting for the signal, continuing to produce more premature responses than before the lesion despite extensive training. This deficit extended to a self-timing task, where rats were required to release a lever within a specific time window following a fixed delay (e.g., 1 s). Bilateral DLS lesions caused a leftward shift in the distribution of self-timed response times. This rise in anticipatory responses was accompanied by reduced movement vigor, including both locomotion and paw reaching—a seemingly paradoxical observation. 

We chose lesions as our perturbation method for this study. Lesions, followed by a recovery period of several days, allow downstream brain areas to restore normal spiking activity, reducing nonspecific effect and better isolating the specific behavioral consequence of losing a brain region (Otchy et al., 2015). The resulting deficits reflect both the immediate and long-term consequences of removing the targeted brain region. While the transient impairment caused by lesions can lead to variable interpretations (Wolff and Ölveczky, 2018), the enduring impairments underscore the critical role of the region in maintaining specific functions (Vaidya et al., 2019). Studies involving lesions in multiple brain regions under the same behavior task can provide valuable insights into the neural basis of complex motor or cognitive functions—for instance, different brain regions may have dissociable roles in a single behavior (Kawai et al., 2015; Dhawale et al., 2021).

Our study primarily targeted the primary MO in MO lesions, but the lesion area also extended to the secondary MO (Fig. 4; Neafsey and Sievert, 1982). While a subset of MO neurons exhibit persistent firing during the delay period (Figs. 2, 3; Narayanan and Laubach, 2006, 2009), the primary lasting effect observed after MO lesions was a mild slowing of RT, on the order of 11.6 ± 4.6% (uMO, mean ± SEM) and 16.2 ± 3.3% (bMO), rather than an increase in premature responses (Martin et al., 1993; Smith et al., 2010). It remains to be determined whether acute inactivation of MO, through methods such as optogenetics, would induce more premature errors in the SRT task (Guo et al., 2015; Galiñanes et al., 2018). Nevertheless, our results suggest that MO was not necessary for waiting but, instead, was helpful for developing fast reactive responses. Although we did not test the role of MO in self-timing task directly, a previous study shows that MO lesions have little effect on press duration learning in a task requiring mice to hold the lever for progressively longer durations (Yin, 2009).

In contrast to MO lesions, bilateral, not unilateral, DLS lesions produced more severe and persistent deficits. Lesioned rats developed a much greater premature error rate, with limited improvement following weeks of training. This behavioral deficit is reminiscent of a previous study using dopamine depletion in rats performing the same task, which also reported a sustained premature response increase (Amalric et al., 1995). We extended this finding to a self-timing task and found that response times shifted toward shorter durations. That is, bDLS-lesioned rats were less able to delay their response until the end of the FP. This deficit was not due to a motor deficit. When we extended the FP from 1 to 1.5 s, we observed a reduction in anticipatory responses before 1 s and a shift in the response distribution to ∼1.5 s (Fig. 8), suggesting that the rats were able to adjust their behavior based on the extended time, which argues against a pure motor impairment (e.g., difficulty holding the lever). Sustained increases or decreases in neural activity related to self-timing behavior have been characterized in the output nucleus of the rat's basal ganglia, the substantia nigra pars reticulata (SNr; Fan et al., 2012). However, how striatal lesions alter these firing patterns remains unexplored. Investigating this could be a crucial step in understanding how striatal lesion affects the brain network underlying timing behavior. Besides the striatum, subthalamic nucleus (STN) lesions (Baunez et al., 1995) also produced a sustained increase in the premature response rate in the same task. The STN receives inhibitory inputs from the external globus pallidus (GPe), which, in turn, is inhibited by the striatum's indirect pathway (Dudman and Gerfen, 2015). It is possible, therefore, that DLS lesion reduced STN activity through disinhibiting the GPe neurons. Further experiments are needed to determine whether the DLS, dopamine, and STN facilitate waiting in series or in parallel. To our knowledge, although lesions of some brain region, including the prelimbic cortex (Risterucci et al., 2003), lead to temporary premature increase, lesions that produce a long-last effect on the premature responses in a RT task are rarely reported.

The compromised waiting suggests a disruption of an internal model that determines when to act. As noted previously, subjects performing the SRT task with the goal of fast responding often anticipate the stimulus timing, improving RT at the cost of emitting some responses irrespective of the stimulus (Ollman and Billington, 1972; Kornblum, 1973). In the “deadline” model (Ollman and Billington, 1972), a behavioral response (e.g., lever release) is emitted either at the deadline or at the time of signal detection, whichever comes first. The deadline is a random variable with a cumulative distribution function (“anticipation function”) that the subject adjusts to optimize performance. Through training, a subject may set the deadline near or beyond the longest FP to avoid many premature errors. The effect of bDLS lesions could thus be interpreted as a disruption of the deadline distribution. In the future, tracking the dynamics of a large ensemble of DLS neurons as the animal learns to wait could provide insight into the neural basis of the deadline model.

Does the increased anticipation seen in bDLS-lesioned rats reflect deficits in temporal processing (MacDonald and Meck, 2004)? That is, lesioned rats became poorer at estimating time. Indeed, some studies have suggested that the dorsal striatum, although not specific to the DLS, is involved in time estimation (Meck, 2006; Gouvêa et al., 2015; Monteiro et al., 2023). For instance, inactivating the striatum reduces the precision of judging the duration of a stimulus (Gouvêa et al., 2015). It is possible that, in our study, bDLS-lesioned rats overestimated time, perceiving short durations as longer and thus responding prematurely in the SRT or self-timing task. Defining timing ability in animals can be challenging, as they often rely on motor or sensory cues to gauge the passage of time (Safaie et al., 2020; Cook et al., 2022; Robbe, 2023). Future studies are needed to specifically test the role of the DLS in interval timing tasks, such as using the peak-interval schedule (Meck, 2006). Preliminary data suggest bDLS lesions did not prevent rats from forming peak-interval response patterns (data not shown).

A slowing of limb movement has been observed following inactivation or lesions in the BG circuits, with a particular focus on the output nucleus, such as the internal segment of the globus pallidus (GPi; Horak and Anderson, 1984; Mink and Thach, 1991; Desmurget and Turner, 2008). In our experiments involving bilateral DLS lesions, we observed a combination of movement slowing and an increase in anticipatory responses, which is puzzling. Several potential explanations could account for this phenomenon. First, rats with DLS lesions may have adopted an earlier response strategy to mitigate the impact of their reduced movement vigor. Similar findings have been reported in different behavioral contexts. In one study, rats had to time their action against a moving treadmill to collect a reward after a fixed interval (Jurado-Parras et al., 2020). Following bilateral striatal lesions, animals moved more slowly than before but adopted a strategy to start moving earlier. This trade-off between movement speed and timing suggests that lesioned rats were more sensitive to the cost of efforts in producing high-velocity movement. One might also compare our results with an observation in which rats were trained to tap a lever twice at a 700 ms interval (Kawai et al., 2015). In that study, bilateral DLS lesions caused the rats to produce shorter intertap intervals, leading to a persistent behavioral impairment (Dhawale et al., 2021). Although impaired motor kinematics due to DLS lesions could play a significant role, the shortening—rather than lengthening—of response intervals in that study resembles the reduction in waiting observed in our study, particularly in rats making self-timed responses. Finally, it is worth noting that previous studies of GPi lesions in monkeys performing visually guided RT movements did not report an increase in premature responses despite limb movement slowing (Horak and Anderson, 1984). This discrepancy may reflect a difference in response strategy or behavioral adaptation between monkeys and rats, such as their differential sensitivities to effort, the difference in lesion sites (striatum vs GPi), or a combination of these and other unknown factors. Further investigations are needed to test the effect of BG output lesions in rats performing SRT or self-timing tasks.

In a recent study where mice categorized the duration of a tone, inhibiting the striatum's indirect pathway disrupted waiting during the sampling period before the second of two tones was presented, suggesting a role in movement suppression (Cruz et al., 2022). It remains to be determined whether striatum lesions in the interval categorization task produce a persistent deficit in movement suppression. After all, the behavioral paradigm utilized in the previous study differs from the RT or self-timing behaviors studied here. It is worth noting that in our study, bDLS lesions did not simply disrupt the rats' ability to hold an action. This was demonstrated in the self-timing task, in which lesioned rats adaptively prolonged their holding time when the FP was extended. Thus, the increased anticipatory responses associated with bDLS lesions are more likely to reflect a cognitive deficit than a motor one.

Finally, one might postulate that since DLS lesions produced a more severe deficit in inhibitory control, there should be a richer neural dynamics during waiting in the DLS compared with the MO. However, this was not the case. In both regions, comparable subsets of neurons fired spikes during the FP, while other subsets were active in response to other task events, such as orienting toward the lever or the reward port. Dimensionality reduction revealed that the overall population dynamics, once projected onto the principal axes, were similar between MO and DLS. This is expected, given that corticostriatal inputs are the main drivers of striatal neurons (Peters et al., 2021). However, MO and DLS are vastly different brain structures, with MO's principal neurons sending excitatory projections to a variety of target regions, including the spinal cord, while DLS's projection neurons inhibit downstream structures through the direct or indirect pathways. In addition, DLS receives dense innervation from dopamine neurons, which likely mediates motor recovery (relearning) when MO is lesioned. Future experiments are needed to examine whether DLS lesions cause irreversible breakdown of neural encoding in MO or downstream motor-related regions, including the SNr which has been implicated in self-guided actions (Fan et al., 2012; Lintz and Felsen, 2016).

In conclusion, our work identifies the DLS as a key brain structure in managing the timing of actions, specifically in tasks requiring a competition between waiting and responding, supporting the role of the basal ganglia in timing-related behaviors (Yin, 2014). These results shed light on how distinct brain regions regulate response timing in both reactive and internally guided behaviors, with implications for disorders of inhibitory control and motor control.
