# Supplementary figures and images for: The Role of Striatum in Controlling Waiting during Reactive and Self-Timed Behaviors
Source: J Neurosci. 2025 Feb 14;45(16):e1820242025. doi: 10.1523/JNEUROSCI.1820-24.2025 (PMC12005370; doi:10.1523/JNEUROSCI.1820-24.2025)

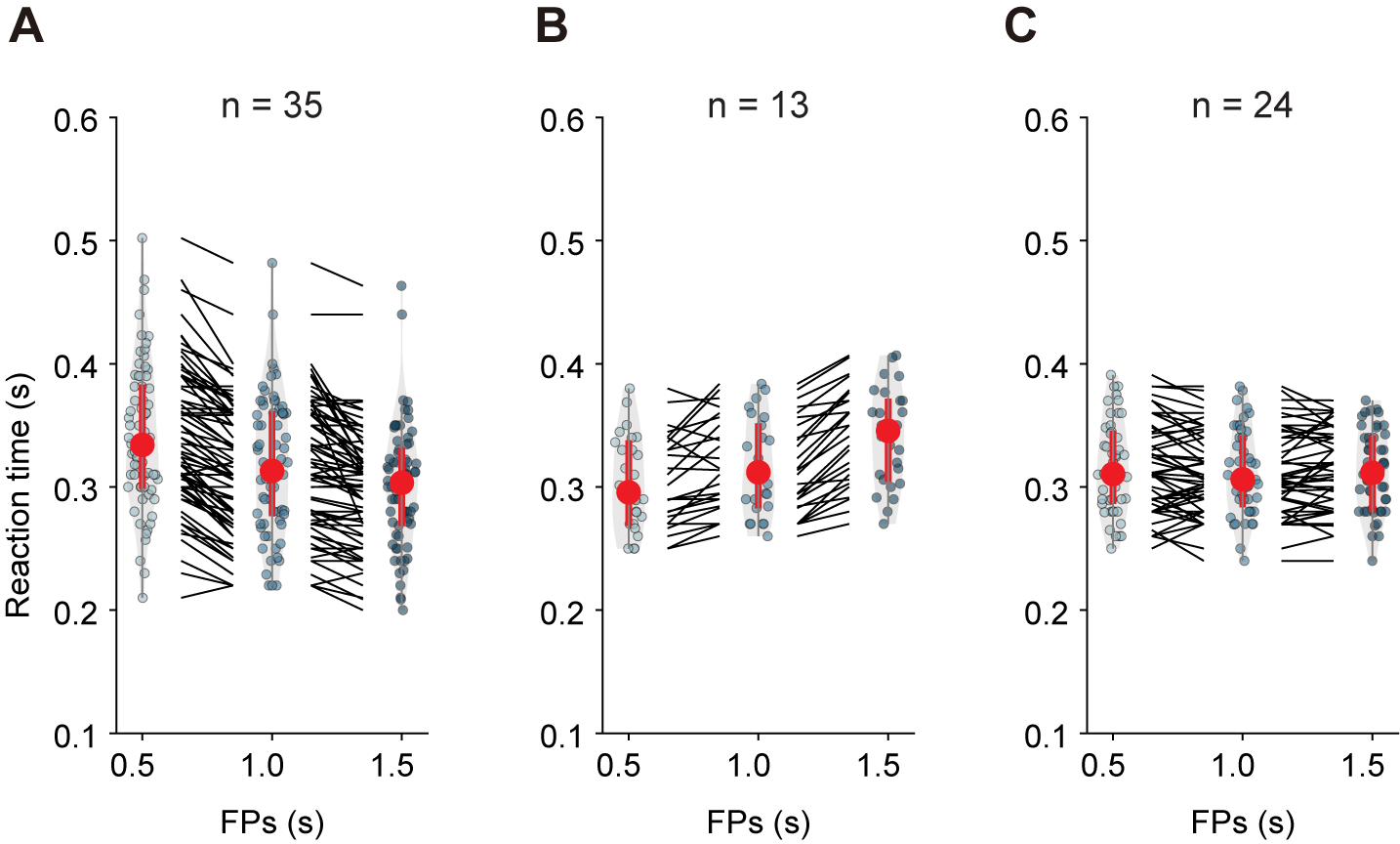

Supplement: Figure 1-1 — Speeding effect. A, Reaction times of 35 rats showed a speeding effect where reaction time decreased with longer FPs. Each dot represents the mean reaction time of a single rat. The red dots represent the median reaction time of all rats. The red lines represent the 25th and 75th percentiles. The whiskers represent the range extending beyond 1.5 times the interquartile range (IQR) above or below the 75th and 25th percentiles. B, Reaction times of 13 rats showed an anti-speeding effect, where reaction time increased with longer FPs. C, Reaction times of 24 rats showed no significant dependence on foreperiod. Statistical testing was performed by fitting a linear model to the reaction time data with FP as a predictor RT ~ 1 + FP. The p-value was extracted from the coefficient estimate for FP. The p-values for all 72 rats were collected, and a Benjamini-Hochberg procedure was applied to compute a critical p-value (0.03389), ensuring the false discovery rate was controlled at less than 0.05. The regression coefficient associated with FP was considered significant if the raw p-value was less than this critical p-value. Download Figure 1-1, TIF file. [file jneuro-45-e1820242025-s004.tif]
